# Irrelevance by inhibition: Learning, computation, and implications for schizophrenia

**DOI:** 10.1371/journal.pcbi.1006315

**Published:** 2018-08-01

**Authors:** Nathan Insel, Jordan Guerguiev, Blake A. Richards

**Affiliations:** 1 Department of Psychology, University of Montana, Missoula, Montana, United States of America; 2 Department of Biological Sciences, University of Toronto Scarborough, Toronto, Ontario, Canada; 3 Department of Cell and Systems Biology, University of Toronto, Toronto, Ontario, Canada; Harvard University, UNITED STATES

## Abstract

Symptoms of schizophrenia may arise from a failure of cortical circuits to filter-out irrelevant inputs. Schizophrenia has also been linked to disruptions in cortical inhibitory interneurons, consistent with the possibility that in the normally functioning brain, these cells are in some part responsible for determining which sensory inputs are relevant versus irrelevant. Here, we develop a neural network model that demonstrates how the cortex may learn to ignore irrelevant inputs through plasticity processes affecting inhibition. The model is based on the proposal that the amount of excitatory output from a cortical circuit encodes the expected magnitude of reward or punishment (“relevance”), which can be trained using a temporal difference learning mechanism acting on feedforward inputs to inhibitory interneurons. In the model, irrelevant and blocked stimuli drive lower levels of excitatory activity compared with novel and relevant stimuli, and this difference in activity levels is lost following disruptions to inhibitory units. When excitatory units are connected to a competitive-learning output layer with a threshold, the relevance code can be shown to “gate” both learning and behavioral responses to irrelevant stimuli. Accordingly, the combined network is capable of recapitulating published experimental data linking inhibition in frontal cortex with fear learning and expression. Finally, the model demonstrates how relevance learning can take place in parallel with other types of learning, through plasticity rules involving inhibitory and excitatory components, respectively. Altogether, this work offers a theory of how the cortex learns to selectively inhibit inputs, providing insight into how relevance-assignment problems may emerge in schizophrenia.

## Author summary

Individuals with schizophrenia have difficulty ignoring ideas and experiences that most people would treat as unimportant. There is evidence that this may be due to changes in neuronal inhibition, suggesting that inhibitory neurons may be involved in learning to ignore irrelevant inputs. By developing a computational model that learns relevance and irrelevance through changes in the strength of feedforward inhibition, we are able to simulate many specific effects of inhibitory neuron dysfunction on behavior. We also show two computational advantages to this mechanism: (1) if relevance is signaled by the level of excitatory activity, then downstream circuits can easily avoid learning from irrelevant stimuli, (2) relevance learning can occur simultaneously with other types of learning. The model therefore offers insight into the relationships between neural inhibition and behavior, including symptoms of schizophrenia.

## Introduction

Many symptoms of schizophrenia can be understood as an inability of the brain to appropriately assign relevance to environmental stimuli and internal representations. Schizophrenic patients exhibit difficulties filtering-out, or gating, irrelevant external stimuli [1, 2, 3, 4, 5, 6, 7], and delusions may also be the product of misattributing relevance (or “salience”) to certain types of internally-generated representations [8]. While many neural explanations have been proposed, convergent evidence points to dysfunction in inhibitory processes within the neocortex. This idea dates back at least to Johnson (1985) [9], who hypothesized that schizophrenia symptoms arise from a failure of feedforward inhibition—i.e. activation of inhibition by a system’s inputs. Circuits for cortical feedforward inhibition are now relatively well defined, and may principally involve fast-spiking, parvalbumin-expressing (PV+) inhibitory interneurons [10, 11, 12, 13]. It is also now well established that PV+ interneurons are compromised in schizophrenia (reviewed by [14, 15, 16, 17, 18]).

Computational models have helped to articulate the link between inhibitory dysfunction and schizophrenia [19, 20, 21]. An important example is work by Vogels & Abott (2007, 2009) [19, 20], which demonstrated how inhibition may serve to selectively gate some representations but not others. A theme of these models is the importance of balanced excitation and inhibition (EI balance) within the network. EI balance has been extensively studied across a range of cortical regions (e.g., auditory cortex [22, 23, 24], somatosensory cortex [25, 26, 27, 28], olfactory cortex [29], visual cortex [30, 31], and frontal cortex [32]). Importantly, EI balance can fluctuate dynamically, and can reflect the expectation of rewards or punishments [33, 34, 35, 36, 37]. Therefore, a better understanding of the relationship between cortical inhibition, reinforcement signals, and relevance coding may be critical to understand schizophrenia.

The goal of the present study is to improve our understanding of how disruptions in neural inhibition could compromise the brain’s ability to ignore irrelevant inputs, as observed in schizophrenia. Three main questions are addressed. First, how might inhibitory neurons learn the relevance of specific input patterns, as defined by the patterns’ ability to predict reward or punishment? Second, how might this learning, and corresponding fluctuations in EI balance, help explain experimentally observed relationships between cortical inhibition and behavior? Third, how might relevance learning in inhibitory neurons fit with other learning mechanisms in cortex, such as category learning? Answering these questions will help explain how inhibitory neurons contribute to the “gating” of inputs, potentially lending insight into how neural dysfunction may result in some symptoms found in schizophrenia.

**Fig 1:**
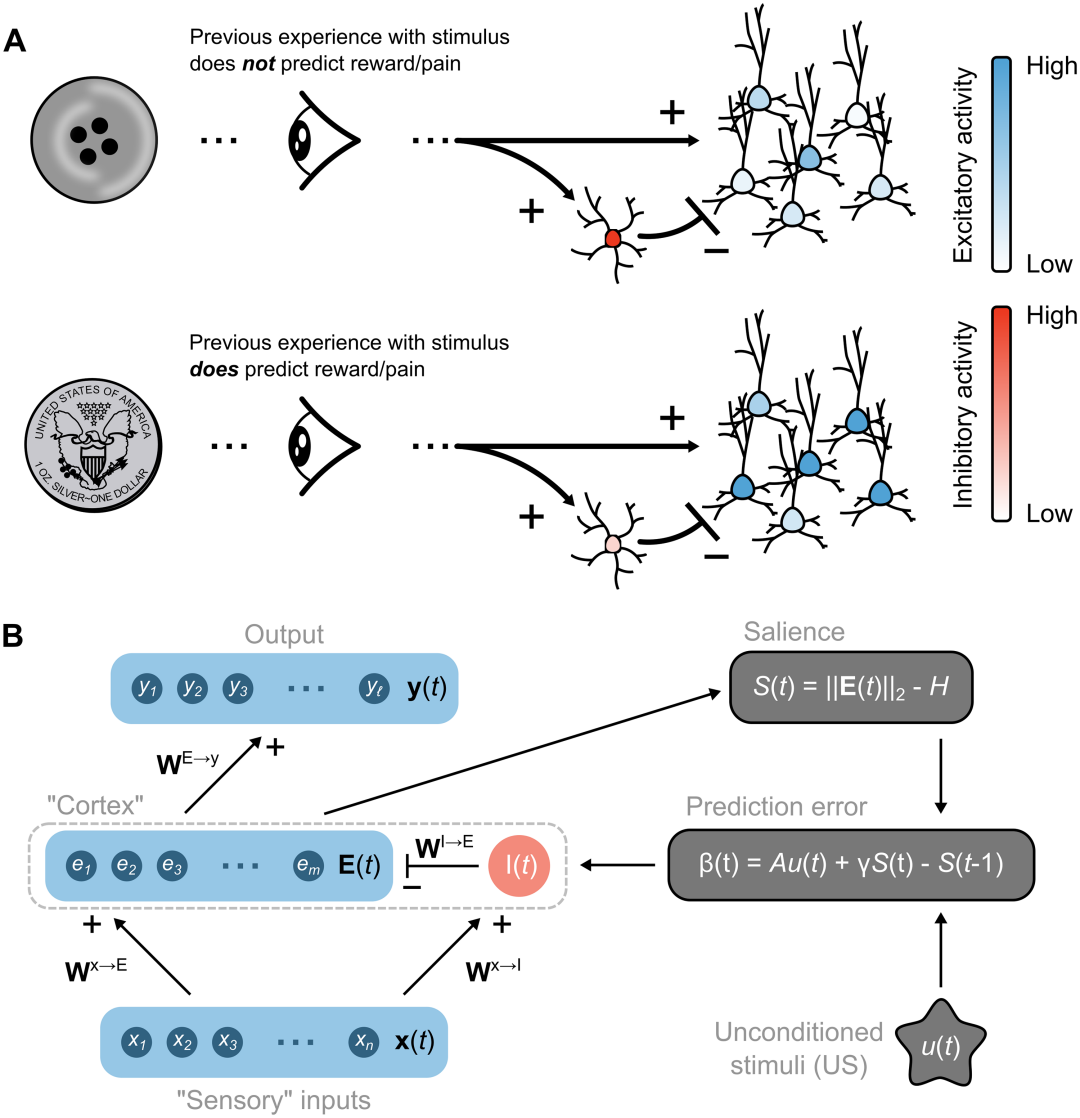
Overview of the proposed relevance code and network model. (**A**) Schematic illustrating the hypothesis that relevance (prediction of reward or punishment) is coded by levels of excitatory neuron output from a network, which is controlled by feedforward inhibition. (**B**) Basic structure of the network model. Left side shows feedforward connections from “Sensory” inputs, through inhibitory (*I*(*t*)) and excitatory (**E**(*t*)) “Cortex” units, with **E**(*t*) units feeding onto an output layer. Right-side shows how the salience signal (*S*(*t*)), computed from the overall level of excitatory unit activity, is combined with signals about environmental unconditioned stimuli (*u*(*t*)) to generate a prediction error that supervises the plasticity of connection weights between Sensory and Cortex layers.

To answer the three questions above, we have developed a neural network model that can learn to ignore specific inputs, but not if inhibition is disrupted. The fundamental proposal in the model is that the overall level of excitation in a cortical circuit signals the temporally discounted expectation of rewards and/or punishments (Fig 1A; [38]). According to this formulation, deviations in EI balance come to represent the network’s estimate of the magnitude of the value signal used in reinforcement learning [39]. By representing relevance using the magnitude of excitatory activity across the population, it is easy for a downstream circuit with a threshold to ignore irrelevant stimuli. Furthermore, this formulation also enables a “multiplexed” code, where the population-level activity represents relevance, while the specific pattern of activity can represent other pieces of information (e.g. stimulus category).

Three sets of simulations are used to demonstrate the explanatory power of the model. The first set of simulations demonstrate the model’s capacity to learn about input relevance/irrelevance, and that, paralleling symptoms of schizophrenia (e.g., [40, 41, 42]), relevance processing is disrupted by impaired inhibition. The second set of simulations use an extended model to show how the proposed relevance code can be used by a downstream circuit to prevent behavioral adaptation to irrelevant stimuli, which we use to reproduce the effects of manipulating inhibition in rodent frontal cortex [43, 44]. The final set of simulations show how relevance learning could occur concurrently with other types of learning, e.g. categorization of input patterns, thereby providing a mechanism to multiplex information about stimulus-relevance and stimulus-identity. Importantly, this model is not meant to provide a comprehensive theory of relevance learning, nor the etiology of schizophrenia, but to offer a computational proof-of-concept for how circuit dysfunction may result in certain, observed behavioral pathologies.

## Results

### Network summary

Our first goal was to develop a simplified neural network model in which feedforward inhibitory processes are involved in learning to ignore a stimulus. We take as an assumption that cortical brain networks, as a default, are relatively more responsive to novel input patterns. We therefore define “learning to ignore” as the process by which a network learns to be less responsive to those stimuli that are not predictive of rewards/punishments. Behaviorally, repeated presentations of a stimulus lead to subjects taking longer to associate that stimulus with a second, valued stimulus—a phenomenon known as latent inhibition [45]. Latent inhibition is known to be impaired in schizophrenia [46, 47, 48, 49]. While we ultimately develop the model into one that exhibits latent inhibition (see “Effect of relevance learning on downstream circuitry”, below), the first step was to build a network that could maintain a high level of responding to a stimulus that predicts the arrival of an unconditioned stimulus (US), while responding less to stimuli that do not make predictions about an US.

The basic structure of the model is illustrated in Fig 1B and described in detail in Methods. Briefly, the input, ‘Sensory’ layer of the network, **x**(*t*) = [*x*_1_(*t*), …, *x*_*n*_(*t*)] (*n* = 1000), drives activity in the ‘Cortex’ layer excitatory units, **E**(*t*) = [*e*_1_(*t*), …, *e*_*m*_(*t*)] (*m* = 800), through a set of positive connection weights, **W**^*x*→*E*^. (For notation purposes, we use bold symbols for all vectors and matrices). The Sensory layer also drives activity in the Cortex inhibitory population unit, *I*(*t*), through positive connection weights **W**^*x*→*I*^. The inhibitory unit divides Cortex excitatory activity through the weight matrix **W**^*I*→*E*^. The inhibitory unit is intended to loosely model the population of cortical fast-spiking inhibitory interneurons, which evidence suggests provide a divisive “blanket” of feedforward inhibition that is synchronized by gap-junctions [50, 51, 52, 53]. Any US (positive or negative) is represented by the variable *u*(*t*) ∈ {0, 1}, which is set to 0 if no reinforcement is present, and 1 if reinforcement is present. Hence, *u*(*t*) is an unsigned reinforcement signal, which simply indicates the presence or absence of a US. Fig 1B also shows Cortical excitatory units acting on a layer of ‘Output’ units. The Output layer was not necessary for the initial simulations of relevance learning, but became essential for recapitulating empirical data and demonstrating multiplexing, as described below.

In order to derive analytical results, we initially relied on a deterministic, rate-based model, i.e. we treated **x**(*t*), **E**(*t*), and *I*(*t*) as rates-of-fire (see Methods). However, in our simulations, we sampled the number of spikes generated by each neuron at each time-step from a Poisson distribution, which introduced stochasticity and, given the short time-steps used, meant that neurons fired only zero or one spike per bin, effectively introducing a threshold non-linearity. Empirically, we found that the behavior which our analytical derivations predicted still applied when Poisson spiking was used in the simulations.

At its core, the ability of the model to learn stimulus relevance or irrelevance depends on feedback from a signaling pathway depicted on the right side (gray boxes) of Fig 1B. The total level of Cortex excitatory unit activity (measured by the norm of **E**(*t*)) is compared against a baseline, homeostatic level (*H*) to compute relevance, or the ‘Salience’ signal (*S*(*t*)):

$$\begin{array}{c} S\left(t\right)={∥E\left(t\right)∥}_{2}-H \end{array}$$ (1)

The goal of learning in our model is to have *S*(*t*) accurately represent the expected future magnitude of unconditioned stimuli, as predicted by current sensory inputs. This would mean that *S*(*t*) would be high for stimuli that predict reward/punishment, and close to zero for stimuli that do not. Put another way, the goal of learning in the model is to have *S*(*t*) come to represent the variable *U*(*t*), which is an unsigned version of the value function from reinforcement learning [39]:

$$\begin{array}{cc} U(t) & =\langle \sum_{i=1}^{\infty }\gamma^{i-1}u\left(t+i\right)\rangle \end{array}$$ (2)

where 0 < *γ* < 1 is a temporal discounting term and 〈⋅〉 indicates the expected value. The formal goal of relevance learning in our model is to have *S*(*t*) be equal to a scaled version of *U*(*t*), i.e. to have *S*(*t*) = *AU*(*t*), where *A* is a scaling variable set to achieve physiologically realistic levels of cortical activity (see Methods). If we can achieve this goal, then the overall level of excitation in the Cortical layer encodes an estimate of how relevant a set of sensory inputs are for predicting reward/punishment. In such a case, stimuli that are predictive of an US will drive higher overall levels of excitatory activity than stimuli that are uninformative regarding an US. A downstream circuit could then use this *S*(*t*) value implicitly or explicitly to drive learning or gate behavioral reactions (we touch on this more below). We note, though, that any downstream circuit that utilized the explicit value of *S*(*t*) itself would require some form of non-linear calculation to compute the vector norm.

From a practical perspective, one way to ensure that *S*(*t*) = *AU*(*t*) is to perform stochastic gradient descent on the squared difference between *S*(*t*) and *AU*(*t*). More precisely, we can update the synaptic weight, $W_{j}^{x\to I}$, from unit *j* in the Sensory layer onto the inhibitory unit using the following learning rule:

$$\begin{array}{c} \begin{array}{cc} W_{j}^{x\to I} & \leftarrow W_{j}^{x\to I}+\alpha \Delta W_{j}^{x\to I}\\ \Delta W_{j}^{x\to I} & =-\frac{\partial {\left(S\left(t\right)-AU\left(t\right)\right)}^{2}}{\partial W_{j}^{x\to I}} \end{array} \end{array}$$ (3)

where *α* is the learning rate. Based on the equations given in the Methods, we derive the following:

$$\begin{array}{cc} \frac{\partial {\left(S\left(t\right)-AU\left(t\right)\right)}^{2}}{\partial W_{j}^{x\to I}} & \propto \beta \left(t\right)x_{j}\left(t\right) \end{array}$$ (4)

where *β*(*t*) is a prediction error term:

$$\begin{array}{c} \beta (t)=Au(t)+\gamma S(t)-S(t-1) \end{array}$$ (5)

This prediction error term corresponds to an unsigned version of the *δ* prediction error term that is common in reinforcement learning [39]. Indeed, this learning update is equivalent to an unsigned version of the temporal difference learning algorithm [39]. It can be shown that the learning algorithm defined by Eq 3 converges when the following condition holds:

$$\begin{array}{c} {∥E\left(t\right)∥}_{2}=H+AU\left(t\right) \end{array}$$ (6)

When taken together with the definition of *S*(*t*) given in Eq 1, we know that if Eq 6 is true, then the goal of having *S*(*t*) = *AU*(*t*) is met.

For most simulations, we updated the Sensory-to-Inhibitory synapses (**W**^*x*→*I*^), as specified in Eq 3. However, the same method of stochastic gradient descent can be applied to any synapses in the network. Therefore, to explore other possible mechanisms for relevance learning, in two other sets of simulations (see Relevance Learning in Methods and Learning to ignore and blocking below) we examined how relevance learning operates when a similar gradient descent rule is applied to Sensory-to-Excitatory (**W**^*x*→*E*^) or Inhibitory-to-Excitatory (**W**^*I*→*E*^) synapses. The equations for these learning updates are provided in Methods.

It should be noted that the model is highly abstract, and makes a number of simplifications for the sake of mathematical tractability. For example, we omit feedback connections between excitatory units in the Cortex layer to focus the present investigation on the hypothesis that plasticity in *feedforward* inhibition can support relevance learning (discussed in more detail in Discussion). Additionally, we generally steer away from being overly specific in identifying brain regions (or networks of regions) and neurotransmitters with the specific computational processes that are modeled. For readability, and general conceptualization, we offer the following approximate mapping between modules in the model and the brain, and discuss the implications of this in more detail in Discussion: “Cortex” is inspired by work in anterior cingulate cortex (in rodents, the medial prefrontal cortex, or mPFC); “Sensory” therefore represents afferents to the anterior cingulate/mPFC; “Output” is modeled in some simulations as the amygdala (detailed below), and in another simulation represents a downstream region of cortex that categorizes stimuli presented to the “Sensory” layer; finally, we think of the salience signal and prediction error as a combination of neuromodulatory inputs and intrinsic homeostatic processes that could, in principle, also engage loops between cortex and sub-cortical systems. A model at this level of abstraction captures only a minor set of the physiological features present in these brain regions, so these interpretations should be judged as semi-agnostic.

Given this framework, and with the ultimate goal of simulating function and dysfunction of behavioral phenomena like latent inhibition, our first goal was to demonstrate whether the model could indeed learn to use *S*(*t*) to represent the relevance of the Sensory inputs for predicting a US.

### “Learning to ignore” can occur with inhibitory interneuron plasticity

The first set of simulations tested whether the model was capable of learning to ignore specific stimuli after repeated presentations. The principle idea is that all novel stimuli are treated as intrinsically salient (high *S*(*t*)), but if a stimulus is not predictive of other valued experiences then the network will learn to reduce its estimate of salience to the level that would be observed if no stimulus were present. The simulation was run using a time step (*dt*) of 20 ms, which was chosen because it approximates the estimated cortical pyramidal neuron membrane time constant [54, 55, 56] and the inter-spike-intervals of fast-spiking basket neurons (and, relatedly, the period of the gamma oscillation). This timestep is also still large enough to prevent Poisson noise from having an undue effect on the gradient calculations. Each unit of the Sensory layer was assigned a baseline activity level to simulate the layer’s response to contextual variables. At the beginning of the simulations, a 60 s adaptation period without stimulus presentations was run, which allowed the synaptic weights to adjust to this baseline.

**Fig 2:**
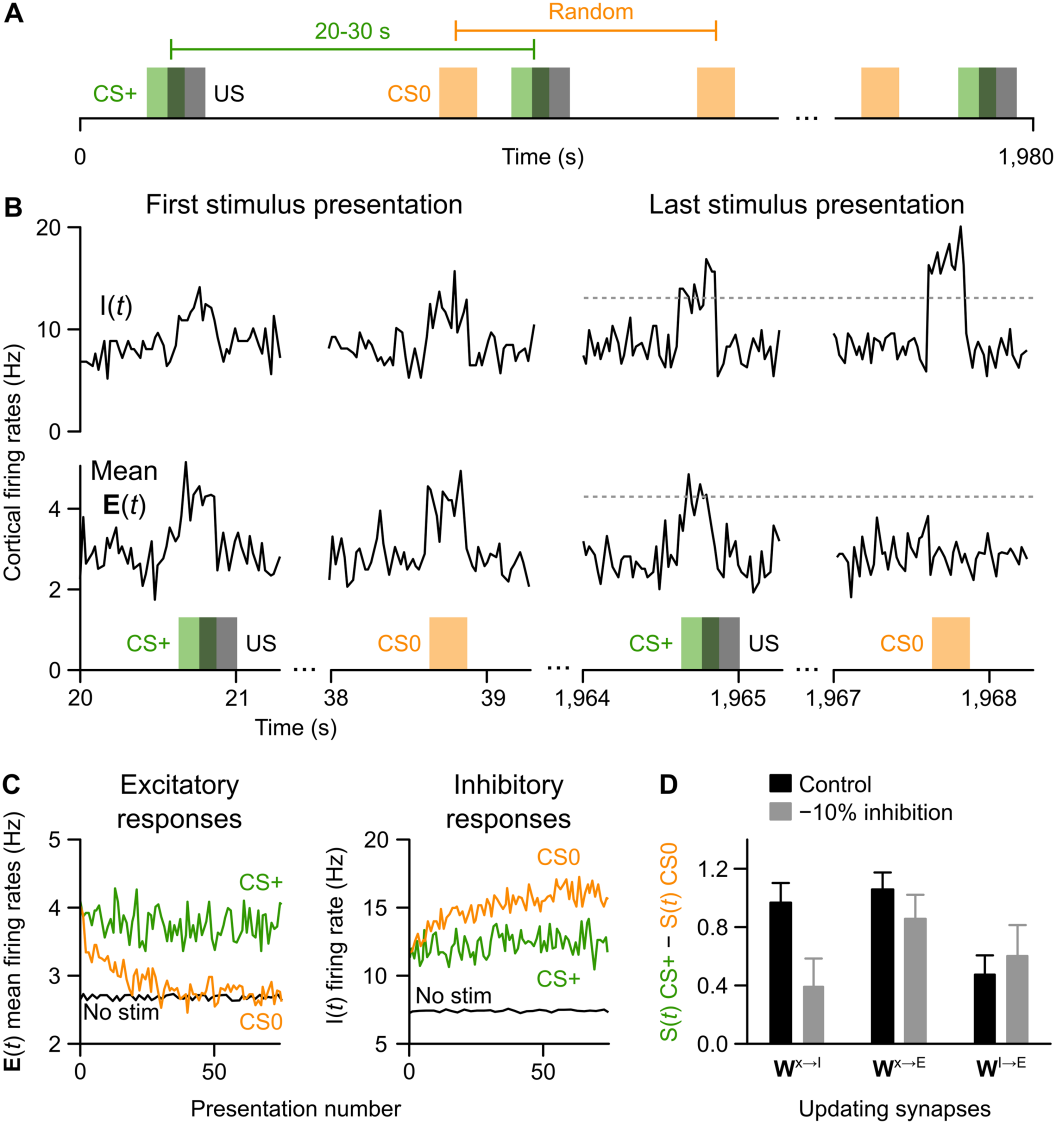
Impaired learning to ignore following disruption to inhibition. (**A**) Illustration of the “learning to ignore” training paradigm. CS+ inputs (green bars) were paired with the US (grey bars), while CS0 inputs (orange bars) were random with respect to the US. (**B**) Average Cortex excitatory unit activity (lower plots) and inhibitory unit activity (upper plots) at simulated, 20 ms time steps in response to unlearned stimuli (left side) compared with the end of a series of repeated presentations (right side). Excitatory responses were initially high to both stimuli, but after learning they increased only in response to the CS+, demonstrating the network has learned to ignore the CS0. (**C**) Averaged excitatory unit (left) and inhibitory unit (right) responses to the CS+ (green) and CS0 (orange) across presentations, as compared with non-stimulus periods (black line). Learning took place over the first 20 trials, after which excitatory responses to the CS0 plateaued to the same level as was observed with no inputs. This was due to increased inhibitory responses to the CS0. (**D**) Salience responses (*S*(*t*)) to the CS+ relative to the CS0 during final presentations are plotted for both control conditions and in simulations of inhibitory dysfunction (means ± STD across 30 model runs). Learning to ignore was impaired with inhibitory neuron disruption only in the inhibitory neuron plasticity model (*W*^**x→I**^).

Following the adaptation period, two different 200 ms long stimuli were presented to the network using independent, inter-trial-intervals of US presentation between 20 and 30 seconds (based on classical conditioning protocols, as in [57, 58]). The stimuli were simulated as increases in the firing rates (20 Hz) of a pre-determined set of Sensory units (10% of the total population). One of the two stimuli, CS+, was consistently paired with a US (by setting *u*(*t*) = 1). The onset of the CS+ preceded the onset of the US by 100 ms, though learning could proceed with different delays between the CS+ and US, if the hyperparameters in the simulation were altered (S1 Fig). In general, the goal of the model was not to capture temporal delay effects, so we did not focus on selecting hyperparameters that reproduced experimental findings on CS-US delay periods. Moreover, as a model with no recurrent dynamics, any ability to account for more interesting temporal phenomena is limited. The other stimulus, CS0, was random in time with respect to the US. The set of Sensory units representing the CS+ was non-overlapping with the set of units representing the irrelevant stimulus, CS0 (Fig 2A). Explanations for the parameters used for connection weights and firing rates are provided in Methods. In general, all firing rate parameters were based on observations made from Ref [38].

During the initial presentations of the CS+ and CS0, the network responded with increased levels of Cortex inhibitory unit activity (Fig 2B, top left panels above colored boxes) and excitatory unit activity (Fig 2B, bottom-left panels). This was due to the increased input from the Sensory layer, **x**(*t*), associated with presentation of either stimulus. As the number of presentations accumulated, there was a selective reduction in the degree to which excitatory units responded to the CS0, to the point that the CS0 was treated as being equivalent to an absence of a stimulus, from the perspective of overall levels of excitation. But, there was no reduction in the degree to which the network responded to the CS+ (Fig 2B, *right*). Thus, the network learned to “ignore” the CS0 (treat it like an absence of stimuli) and not the CS+. Fig 2C illustrates the gradual decrease in excitatory unit population responses to the CS0 (*left*) and the corresponding increases in the Cortex inhibitory unit (*I*(*t*)) response (*right*). S2 Fig shows the distribution of excitatory unit firing-rates across the simulation and the final distribution of the trained synaptic weights.

The increased responsiveness of the Cortex inhibitory unit to the CS0 over presentations was due to the gradually increased connection weights between the units of the Sensory layer and the Cortex-inhibitory unit (**W**^*x*→*I*^), caused by the learning rule. We next examined whether the same patterns could be observed using other model versions, in which synapses either between Sensory and Cortex-excitatory units (**W**^*x*→*E*^), or between Cortex-inhibitory and excitatory units (**W**^*I*→*E*^) were modified. This comparison allowed us to assess how each model responds to disrupted inhibition (see Methods): if current theories of impaired inhibition in schizophrenia are correct [17], then disrupting inhibition in our model should produce impairments in the ability to learn to ignore irrelevant stimuli, as is observed in schizophrenic patients [46, 47, 48, 49].

The results of these tests are described in Fig 2D. Both **W**^*x*→*I*^ plasticity and **W**^*x*→*E*^ plasticity models exhibited much better learning of relevant versus irrelevant stimuli, indicated by the salience signal (*S*(*t*)) during the CS+ relative to CS0, compared with the **W**^*I*→*E*^ plasticity model. Disrupted inhibition only eliminated the ability to learn to ignore in the **W**^*x*→*I*^ plasticity model. Differences in how the model types responded to disrupted inhibition could be assessed statistically: even ten repetitions of the simulation was more than sufficient to demonstrate an interaction effect between model type and inhibitory manipulation (two-way ANOVA, type × manipulation: *F*_(2, 54)_ = 23.97, *p* = 3.6 × 10^−8^; one-way ANOVA comparing the disrupted inhibition conditions: *F*_(2, 27)_ = 14.87, *p* = 4.4 × 10^−5^, multiple comparisons between all groups significantly different using a Bonferroni correction).

The use of a single unit to simulate all feedforward inhibition is obviously not biologically realistic, and evidence suggests that models with a single inhibitory input cannot capture the true complexity of disruptions to EI balance that occur in some neurological disorders [59]. Moreover, the effects of manipulating inhibition may depend on detailed excitatory-inhibitory interactions [60]. Hence, one potential concern is that our results would not be reproduced with a more realistic inhibitory network, or even with multiple inhibitory interneurons. However, because our model does not include recurrent excitation and feedback inhibition, we effectively have a built-in level of excitatory stability, so the use of a single inhibitory unit may be inconsequential for our specific study. Indeed, analytically, we find that similar results hold when *I*(*t*) is treated as a population (**I**(*t*) = [*i*_1_(*t*), …, *i*_*k*_(*t*)], *k* = 500). To confirm this, we also ran simulations with a more realistic population of inhibitory neurons, rather than a single unit, and we found the same pattern of learning to ignore as occurred with a single inhibitory unit (S3 Fig). Thus, for our particular study, the use of a single inhibitory unit did not affect the results. More detailed models are likely to be very important for understanding cortical dynamics and EI balance [59, 60], but they are not required to understand or examine the basic relevance learning mechanism that we propose here.

These simulations on “learning to ignore” offer a first step toward a more complete model that links behavioral symptoms in schizophrenia, such as latent inhibition, with inhibitory neuron dysfunction. Given the model’s specific set of assumptions and simplifications, a **W**^*x*→*I*^ plasticity model offers the best fit to make this link. This is not to suggest that plasticity of synapses onto inhibitory neurons is impaired in schizophrenia. Rather, it suggests that if real cortical networks rely on plasticity in feedforward inhibitory synapses for learning to ignore stimuli, then the causal link between inhibitory neuron dysfunction and irrelevance learning impairments in schizophrenia can be explained. Hence, if we take inhibitory impairment to be a part of schizophrenia, then our results predict that relevance learning in the cortex may be mediated by plasticity of synapses onto inhibitory neurons. The next step was to examine whether other known impairments in relevance learning in schizophrenia could be captured by our model.

### Inhibitory interneuron plasticity can explain blocking data

**Fig 3:**
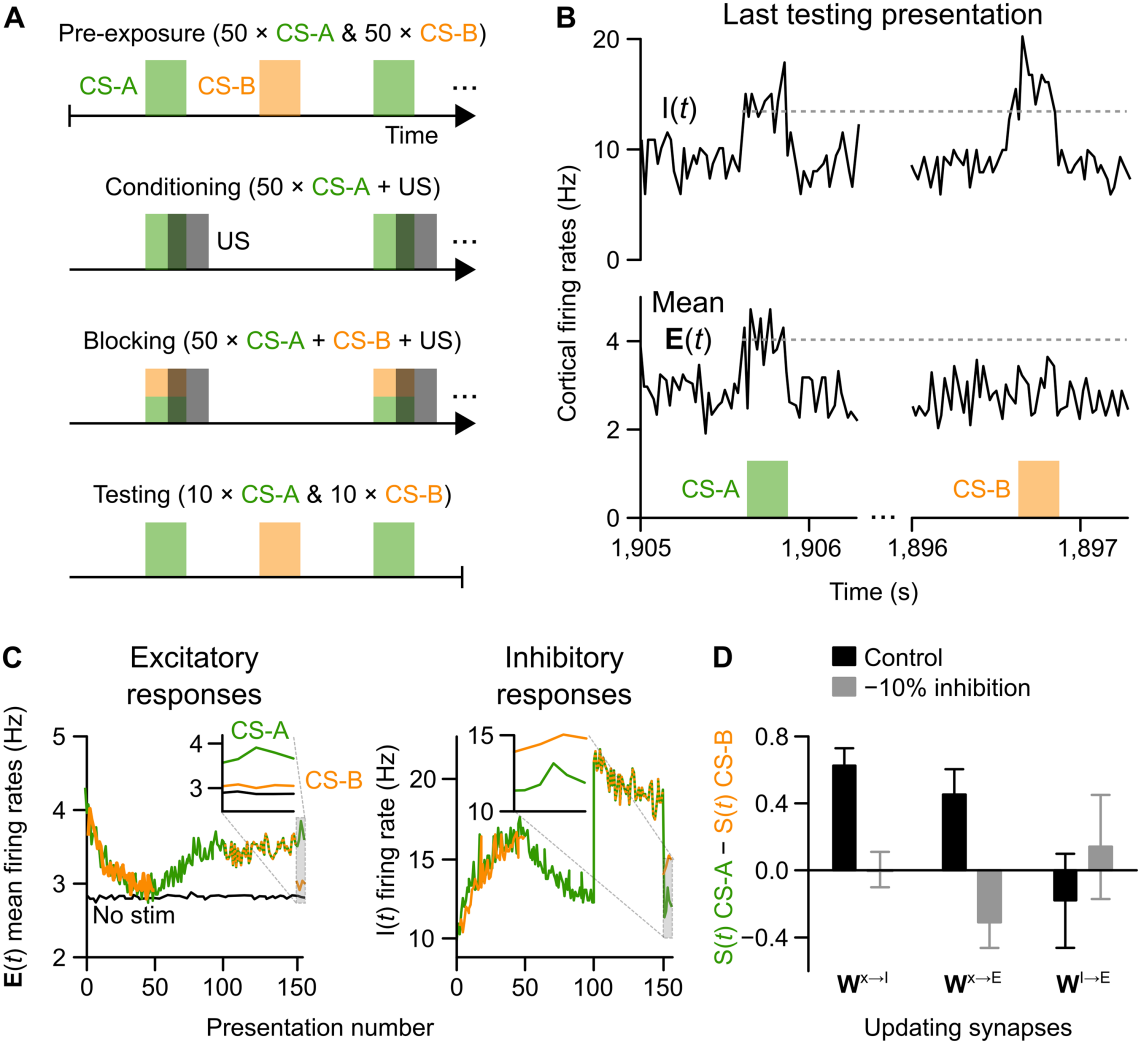
Demonstration of blocking and its impairment following inhibitory disruptions. (**A**) Illustration of the blocking paradigm: the model was first habituated to two stimuli (CS-A, CS-B; Pre-exposure), the CS-A and a US were then repeatedly presented at partially overlapping times (Conditioning), both CS-A and CS-B were then presented with the US (Blocking), followed by independent presentations of CS-A and CS-B (Testing). (**B**) Excitatory (lower plots) and inhibitory (upper plots) unit activity over 20 ms bins show the networks response to CS-A (left) and CS-B (right) at the end of the blocking paradigm. In spite of CS-B being paired with the US, the “blocked” stimulus did not elicit increased activity among excitatory units. (**C**) Excitatory (left) and inhibitory (right) unit responses to CS-A and CS-B over trials. Test epochs are expanded in insets. (**D**) Excitatory responses to CS-A relative to CS-B at the end of the test epoch are plotted in control simulations and simulations with dysfunctional inhibition (means ± STD across 30 model runs). The inhibitory neuron plasticity model (***W***^**x→I**^) showed a loss of the blocking effect when inhibition was disrupted; unexpectedly, the excitatory neuron plasticity model (***W***^**x→E**^) exhibited a reversal of the blocking effect; i.e., CS-B was learned more strongly than CS-A.

Another well-established relevance learning phenomenon is “blocking”, in which one stimulus that has been previously reinforced can occlude learning for another reinforced stimulus [61]. Blocking is also known to be affected in schizophrenia [40, 41, 42]. To examine whether the model exhibited blocking, a standard blocking protocol was simulated, as illustrated in Fig 3A. Two different conditioned stimuli (CS) were presented to the network, CS-A (non-blocked) and CS-B (blocked). As with the previous simulation, each stimulus was simulated as an increase in firing rate of a non-overlapping set of Sensory units (10% of the population). The difference between the non-blocked, CS-A, stimulus and the blocked, CS-B, stimulus is that CS-A was conditioned alone with the US (following habituation pre-exposures) while CS-B was conditioned only when paired with CS-A (following pre-exposures and CS-A conditioning). When this type of protocol is used in either rodent (e.g., [61, 62]) or human (reviewed by [63]) experiments, it leads to CS-A being recognized as relevant for reward/punishment, but CS-B being judged irrelevant. The blocking effect was measured in the model by comparing the response of the excitatory unit population to CS-A and CS-B during the final test sessions (Fig 3B, also Fig 3C *inset*). As predicted, the model exhibited the basic blocking effect seen in people and animals, with CS-A generating a large excitatory response and CS-B generating a small one (Fig 3C, *left inset*). Because learning was supported by inhibitory neuron plasticity (in this case, the ***W***^**x→I**^ plasticity model), changes in population responses to both CS-A and CS-B over stimulus presentations paralleled changes in inhibitory neuron responses (Fig 3C, *right inset*). Notably, a strong increase in inhibitory neuron activity was observed during the “blocking” phase of conditioning, reflecting feedforward inhibition compensating for both stimuli being presented simultaneously (Fig 3C, *presentations 0-50*). We note that this is also consistent with observations of increased fast-spiking neuron activity during stimulus presentations and movement [38].

As with the “learning to ignore” simulation, blocking and the effect of inhibitory disruptions were tested in different versions of the models, defined by which synapses (**W**^*x*→*I*^, **W**^*x*→*E*^, **W**^*E*→*I*^) were plastic. Consistent with our predictions, the blocking effect was eliminated in the **W**^*x*→*I*^ plasticity model, following even a 10% disruption of inhibition (Fig 3D, *left bars*). Blocking was also observed in the **W**^*x*→*E*^ model; however, in this model version an unexpected “reverse blocking” effect was observed following inhibitory disruptions (Fig 3D, *middle bars*). This was likely due to the learning mechanism becoming over-active following inhibitory disruptions, leading to a reduction in synaptic weights corresponding with CS-A presentations (regardless of it being paired with the US). No blocking effects could be obtained in the **W**^*I*→*E*^ model (Fig 3D, *right bars*). Once again, these results could be judged statistically, with 10 repetitions more than sufficient to reveal an interaction between model type and manipulation (two-way ANOVA, *F*_(2, 54)_ = 44.08, *p* = 10^−12^, one-way ANOVA of disrupted inhibition condition: *F*_(2, 27)_ = 12.3, *p* = 2.0 × 10^−4^, with multiple comparisons test with Bonferroni correction showing a significant difference between **W**^*x*→*I*^ and **W**^*x*→*E*^ models).

Similar to the results from the “learning to ignore” simulations, these simulations demonstrate that there is potentially a relatively straightforward link between impaired blocking effects in schizophrenia and inhibitory neuron dysfunction when relevance is mediated by plasticity of synapses onto inhibitory interneurons. Both sets of simulations were built on the empirically-based assumption that “relevance” is coded by increased excitatory neuron activity in the network. The next step was to expand the model to examine whether this code for relevance could be used by downstream circuits, to recapitulate experimental effects of manipulating inhibition in cortex.

### Effect of relevance learning on downstream circuitry

In order to simulate behavior, it was necessary to demonstrate how the output of the Cortex layer, and in particular the relevance signal, *S*(*t*), might be used by an efferent region that directly controls behavioral output. A downstream circuit should be able to use *S*(*t*) to differentially respond to relevant versus irrelevant stimuli, in that relevant stimuli should drive more learning and be associated with increased behavioral responses. A simple way to implement this is by use of a threshold mechanism, such that only activity patterns with sufficiently high levels of excitatory activity can drive a behavioral output. Based on our interest in simulating phenomena like latent inhibition, in which relevance impacts not only behavior, but also learning, we hypothesized that a threshold could be used not only to drive activity in an efferent network, but also to drive learning. Our next step was to provide a proof-of-concept for this idea. Since many of the rodent studies in learned irrelevance and latent inhibition use fear conditioning, our efferent Output layer was designed to loosely represent the mammalian amygdala, and the levels of ‘Amygdala’ unit activity were equated with fear expression.

The Amygdala output layer activities, **y**(*t*) = [*y*_1_(*t*), …, *y*_*ℓ*_(*t*)] (Fig 1B, top layer), were modeled as a competitive network [64] with *ℓ* = 10 units receiving inputs **z**(*t*) = [*z*_1_(*t*), …, *z*_*ℓ*_(*t*)] that were driven by Cortex excitatory activity via an *ℓ* × *m* synaptic weight matrix, **W**^*E*→*y*^:

$$\begin{array}{c} \begin{array}{cc} z_{i}\left(t\right) & =\sum_{j}W_{ij}^{E\to y}e_{j}\left(t\right)-\theta \\ y_{i}\left(t\right) & =\{\begin{array}{cc} z_{i}\left(t\right)+0.5u\left(t\right) & \text{if}\;z_{i}\left(t\right)>z_{j}\left(t\right)\text{,}\;\forall j\neq i\;\text{and}\;\left(z_{i}\left(t\right)\geq 0\;\text{or}\;u(t)>0\right)\\ 0 & \text{otherwise} \end{array} \end{array} \end{array}$$ (7)

where *θ* = *H*/4 is a threshold variable.

What Eq 7 says is: (i) the Amygdala layer is silent unless at least one neuron’s input passes the threshold defined by *θ* or an US is present, and (ii) only one unit in the Amygdala layer can be active at any point in time, i.e. it is a “winner-takes-all” circuit. We use this “winner-takes-all” formulation due to experimental evidence for competitive coding in the Amygdala [65, 66], and because it allows us to derive an analytical guarantee regarding the behavior of the Amygdala layer (see below).

In-line with standard competitive learning methods [64], we update the synapses onto the Amygdala units with the following update rule:

$$\begin{array}{c} \begin{array}{cc} W_{ij}^{E\to y} & \leftarrow W_{ij}^{E\to y}+\alpha_{y}\frac{u\left(t\right)y_{i}\left(t\right)}{{∥E\left(t\right)∥}_{2}}\Delta W_{ij}^{E\to y}\\ \Delta W_{ij}^{E\to y} & =\frac{e_{j}\left(t\right)}{{∥E\left(t\right)∥}_{2}}-W_{ij}^{E\to y} \end{array} \end{array}$$ (8)

where *α*_*y*_ is the learning rate. Note also that the weights $W_{ij}^{E\to y}$ are rescaled after every update such that $\sum_{j}W_{ij}^{E\to y}=1$ (see Methods).

Importantly, we can use the formulation of $\Delta W_{ij}^{E\to y}$ to analytically demonstrate that there is a *θ* for which the Amygdala will only respond to a given sensory input if that input is, or has been, paired with an US. First, we note that according to Eq 8, the Amygdala does not learn if there are no units that pass threshold (i.e. if *y*_*i*_(*t*) = 0 ∀*i*) and no US (i.e. *u*(*t*) = 0). Second, the strength of input to a given Amygdala neuron, *i*, is determined by the dot product $W_{i}^{E\to y}\cdot E\left(t\right)$, where $W_{i}^{E\to y}$ is the set of synapses onto Amygdala neuron *i*. Finally, when a given neuron in the Amygdala, *i*, always “wins” (*z*_*i*_(*t*) > *z*_*j*_(*t*) ∀*j* ≠ *i*) in response to excitatory population vectors sampled from the set $E^{i}\left(t\right)=\left[e_{1}^{i}\left(t\right),...,e_{m}^{i}\left(t\right)\right]\in E^{i}$, then the update rule in Eq 8 will push the synaptic weights for *i* to meet the following condition:

$$\begin{array}{c} W_{ij}^{E\to y}={\left\langle \frac{e_{j}^{i}\left(t\right)}{∥E^{i}\left(t\right){∥}_{2}}\right\rangle }_{E^{i}} \end{array}$$ (9)

where ${\langle \cdot \rangle }_{E^{i}}$ denotes expectation over elements of $E^{i}$. In other words, competitive learning in the Amygdala will encourage the synaptic weight vector for unit *i*, $W_{i}^{E\to y}$, to be a normalized version of the mean of the set of excitatory activity vectors that it “wins”, $E^{i}$. As the unit’s synapses are pushed in this direction, the dot product $W_{i}^{E\to y}\cdot E^{i}\left(t\right)$ will generally increase. Hence, we can assume that inputs to the Amygdala units are initially small, but increase over learning.

Moreover, thanks to the relevance learning that is occurring in the Cortical excitatory population, we can make a more explicit guarantee about Amygdala responses. Consider the case where unit *i* “wins” for a given excitatory input pattern $E^{\prime }=\left[e_{1}^{\prime },...,e_{m}^{\prime }\right]\in E^{i}$. After Amygdala learning has converged, Eqs (7) and (9) tell us that in the absence of an US (*u*(*t*) = 0), the input to unit *i* in response to **E**′ is given by:

$$\begin{array}{c} \begin{array}{ll} z_{i}\left(t\right) & =\underset{j}{\sum }{\langle \frac{e_{j}^{i}}{∥E^{i}\left(t\right){∥}_{2}}\rangle }_{E^{i}}e_{j}^{\prime }-\theta \\ & \leq \underset{j}{\sum }\frac{e_{j}^{\prime 2}}{∥E^{\prime }{∥}_{2}}-\theta \\ & =∥E^{\prime }{∥}_{2}-\theta \end{array} \end{array}$$ (10)

Eq 10 tells us that when no US is present, then *z*_*i*_(*t*) is bounded by ∥**E**′∥_2_ − *θ*. When we consider that relevance learning in the Cortex layer will scale ∥**E**′∥_2_ to be close to *H* for irrelevant sensory inputs, and close to *H* + *A* for relevant sensory inputs, we know that:

$$\begin{array}{c} \begin{array}{cc} z_{i}\left(t\right) & \leq \{\begin{array}{c} H-\theta \;\text{if}\;\text{irrelevant}\\ (H+A)-\theta \;\text{if}\;\text{relevant} \end{array} \end{array} \end{array}$$ (11)

thus, we know there exists a threshold *H* < *θ* < (*H* + *A*) for which the Amygdala can respond only to relevant stimuli. In practice, we find that the *z*_*i*_(*t*) are much lower than *H* for most stimuli, including relevant stimuli, since the weights rarely converge to perfect alignment with a given stimulus pattern. From searching the hyperparameter space we found that a threshold of *θ* = *H*/4 was best for distinguishing relevant and irrelevant stimuli, and this value was used in our simulations.

**Fig 4:**
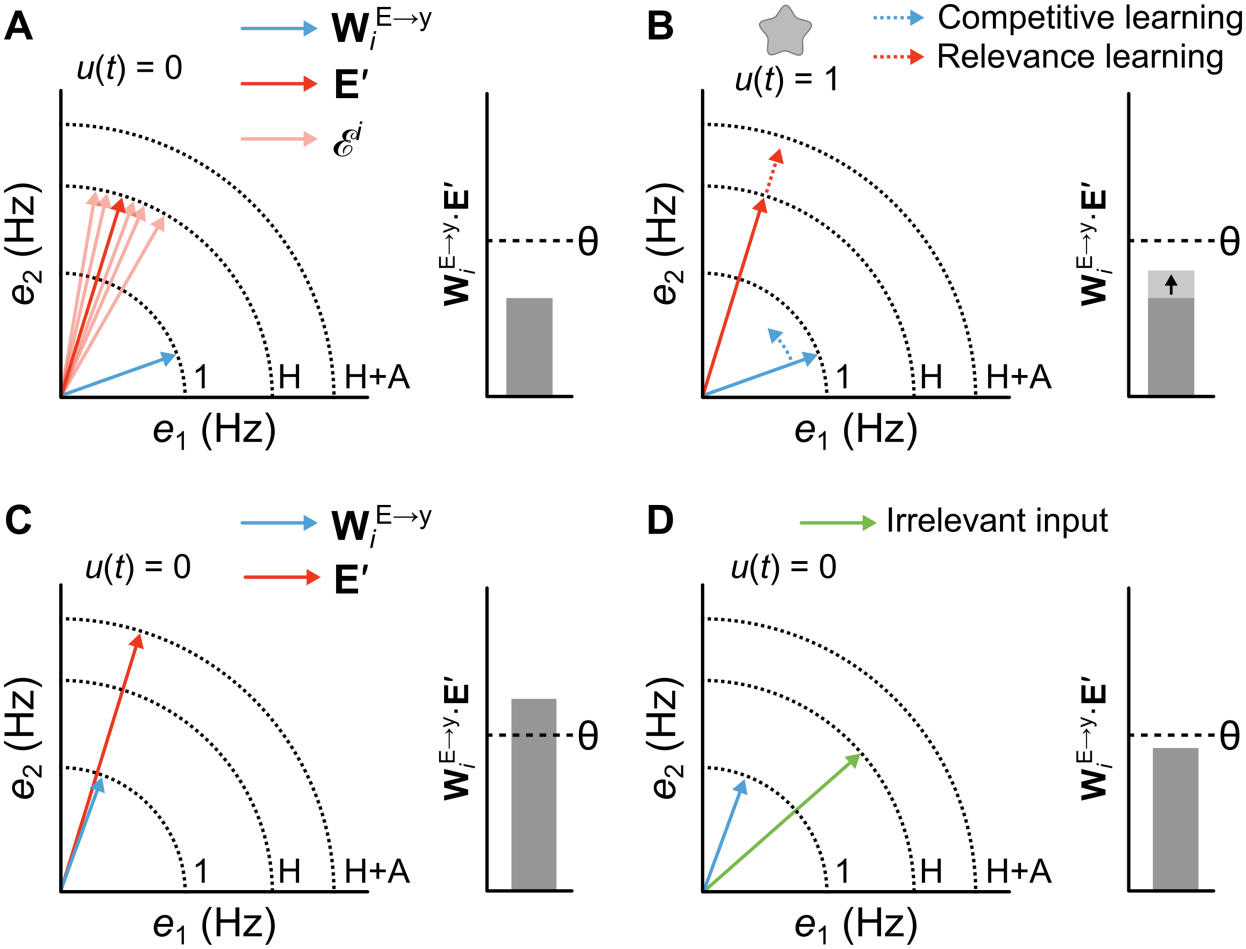
Illustration of interaction between relevance learning and competitive learning. (**A**) Abstract depiction of a network after it is familiarized with stimuli but before it has been reinforced. Left plot depicts a state space with two sets of vectors: red arrows represent a set of activity patterns in the excitatory units, $E^{i}$, blue arrow represents the synaptic weight matrix between these units and an “output” unit *i* in the Amygdala, $W_{i}^{E\to y}$. The dark red arrow represents a prototypical or average state vector $E^{\prime }\in E^{i}$. Right bar plot shows the input level of unit *i*, which is computed as the dot product of the the weight matrix and activity vector of the input units: $W_{i}^{E\to y}\cdot E^{\prime }$. In this case the stimuli are not novel, so all of the associated state vectors in the input units have norms close to the homeostatic constant *H* (lengths of red arrows are approximately *H*). Also, since the weights $W_{i}^{E\to y}$ are not yet trained, they are poorly aligned with $E^{\prime }\in E^{i}$, resulting in activity of *i* being lower than threshold *θ*. (**B**) The same plots as in **A** during learning. When **E**′ is paired with reinforcement (*u*(*t*) = 1), both relevance learning and competitive learning occur. Competitive learning pushes the weight vector $W_{i}^{E\to y}$ in the direction of the mean of input vectors, $E^{i}$ (blue dotted arrow). Meanwhile, relevance learning increases the norm ∥**E**′∥_2_ towards *H* + *A* (red dotted arrow). Although not shown here, the strength of competitive learning depends on the length of the activity state vector; i.e., learning will be stronger for novel or already-salient stimuli. (**C**) As previous, following combined competitive learning and relevance learning. The dot product, $W_{i}^{E\to y}\cdot E^{\prime }$ now exceeds threshold *θ*. This is thanks to both the alignment of the vectors from competitive learning and the increase in the length of **E**′ by relevance learning. Now *i* will become active in response to **E**′ even without an US. (**D**) The same state space is plotted with a vector depicting a different activity state (green arrow) evoked by a stimulus that has been familiarized but not reinforced. The poorer alignment between this new state and the weight matrix coupled with the shorter length for the input vector will yield a lower $W_{i}^{E\to y}\cdot E^{\prime }$ that does not exceed threshold, and thus fails to evoke a response.

To summarize the importance of this result: if no US is present and no training has occurred, then it will be likely that *y*_*i*_(*t*) = 0 ∀*i*, and learning will not occur (Fig 4A). If an US is paired with **E**′, then learning will occur (Fig 4B), particularly if the inputs are novel or already learned to be relevant, because the competitive learning algorithm will make $W_{i}^{E\to y}$ more similar to **E**′. If inputs are not novel or learned to be relevant, then fear learning will take place more slowly, with the competitive learning algorithm taking hold as relevance learning increases the norm ∥**E**′∥_2_ to be closer to *H* + *A*. The increased norm in one layer, and competitive learning changes taking place in the next, increase the dot product $W_{i}^{E\to y}\cdot E^{\prime }$, making it more likely that ∃*i* such that *y*_*i*_(*t*) > 0, even when no US is present (Fig 4C). In this way, we can guarantee that the Amygdala layer only learns and responds to stimuli that are currently being paired with an US or were previously paired with an US.

To put this result in more general terms, we have provided a proof-of-concept for the claim that *if stimulus relevance is encoded using the overall level of excitatory activity in a population, then it is possible for an efferent region to react and learn only in response to relevant stimuli*. We demonstrated this using a simulation implementing a “learned irrelevance” paradigm. This showed that associative learning is slower if a stimulus has previously been learned as being irrelevant than if it has not (S5 Fig).

Although we haven’t explored the use of alternatives to the competitive learning algorithm implemented here, the same principle should apply to any mechanism that uses a threshold and some form of learning that aligns input vectors and synaptic weight vectors. As such, we consider this to be a general, novel insight from the model: not only can relevance learning be implemented using feedforward inhibition to control the overall level of excitatory activity, such an implementation makes it natural for downstream circuits to ignore irrelevant stimuli. In this way, we can gain new insight as to why manipulations of inhibition in cortical afferent regions to the Amygdala can alter animal behavior in fear learning tasks.

### Inhibitory relevance-learning network with amygdala module recapitulates effect of pharmacological manipulations on latent inhibition

**Fig 5:**
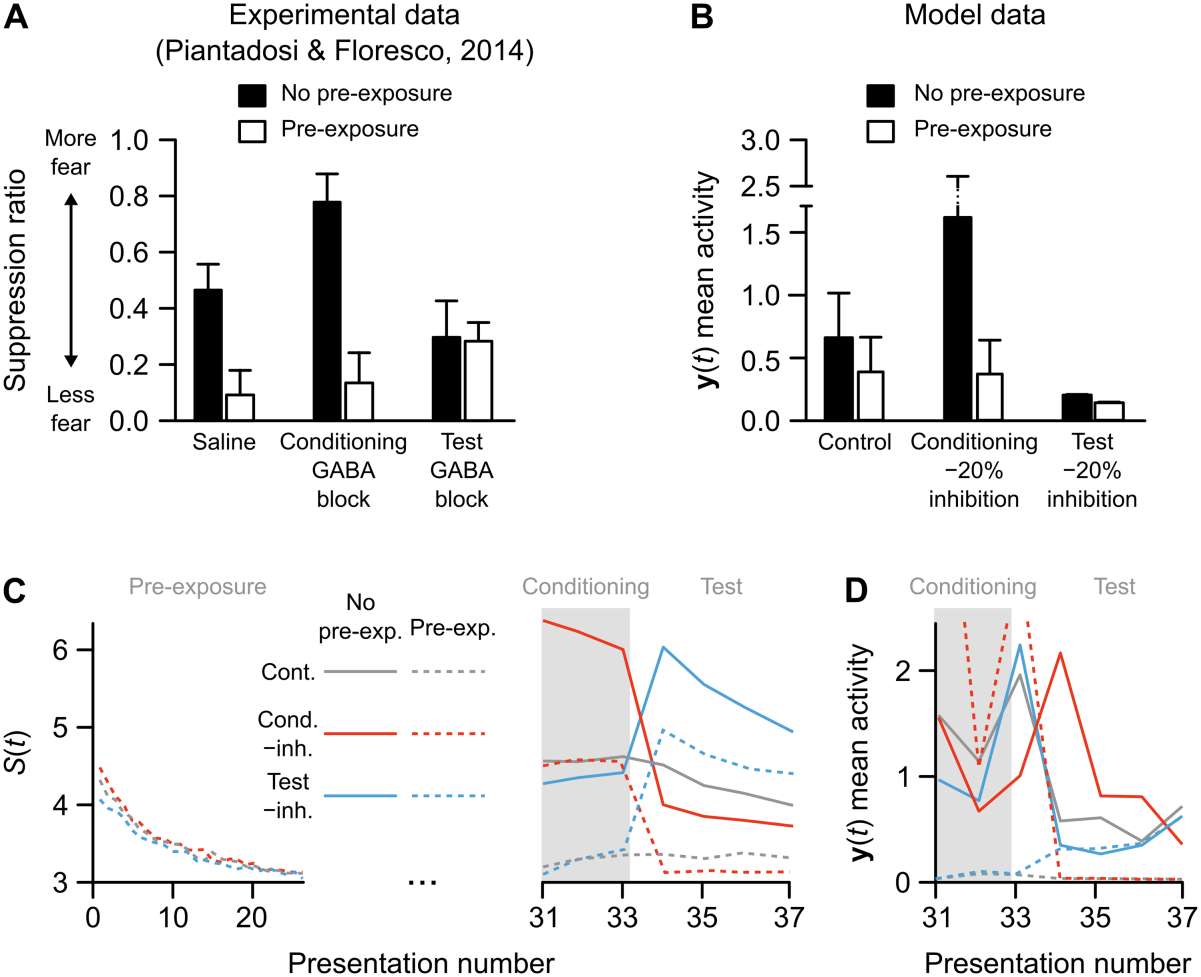
Simulation of experimental data on rodent latent inhibition. (**A**) Fear expression in rats in a latent inhibition paradigm in which animals were either pre-exposed (black bars) or not pre-exposed (white bars) to the conditioned stimulus, and treated with either saline, a GABA-A antagonist during conditioning, or a GABA-A antagonist during testing (reproduced by hand from [43]). (**B**) Data from the model simulation of the same latent inhibition paradigm. Bars show median activity across 30 model runs (errorbars are 90% CI generated by bootstrapping 5-sample medians), of the average Amygdala layer activity during the final (test) stimulus presentations. (**C**) Cortex excitatory unit activity in response to stimuli across trials in an example run of the model. The downward curve during the first 30 presentations shows that the network learned to ignore the CS in all simulations with CS pre-exposures. The activity during conditioning and test periods shows the combined impact of relevance learning and impaired inhibition. (**D**) Amygdala activity levels in an example run of the model over trials with Conditioning and Testing epochs (as in the right-side panel of part C). Test period activity shows a pre-exposure effect in the control condition (solid versus dashed gray lines). This is amplified when inhibition is disrupted during conditioning (solid versus dashed red lines) but was lost when inhibition was disrupted during test (solid versus dashed blue lines).

To determine how relevance learning and our Amygdala circuit interact to produce behavior we simulated experimental studies that link EI balance in cortex to relevance learning and fear conditioning [43, 44]. Our first set of simulations with the Amygdala layer examined the findings of Piantadosi & Floresco (2014) [43]. Their study showed that a GABA-A receptor antagonist, applied to the medial prefrontal cortex (mPFC), can have different effects on latent inhibition when applied at different phases of the learning protocol. As stated previously: latent inhibition refers to the phenomenon wherein it is harder to associate a stimulus with a reinforcer if a subject has previously been exposed to that stimulus. In the study by Piantadosi & Floresco (2014), animals were separated into two groups: those that received pre-exposures to a CS and those that had no pre-exposure. When the CS was subsequently paired with a footshock, the pre-exposure group was less likely to learn the fear association compared with the no pre-exposure group (i.e. the animals exhibited latent inhibition). Importantly, the authors found that blocking GABA-A receptors had different effects if done during the conditioning period or during the test: GABA-A antagonists infused during conditioning amplified latent inhibition, whereas infusions during testing disrupted latent inhibition (Fig 5A). We examined whether our model would exhibit a similar pattern of responses. To determine this, the experiments were simulated using a 20% reduction in inhibition to mimic blockade of GABA-A receptors (see Methods).

As with previous simulations, stimulus presentation was modeled as an increase in baseline firing rate to 20 Hz across a pre-determined set of Sensory units (10% of all units; i.e., 100 units), the timestep used was 20 ms, and 1000 Sensory, 800 Cortex, and 10 Amygdala units were used.

The model showed a similar pattern of results as observed by Piantadosi & Floresco (2014), with simulated GABA-A blockade increasing the latent inhibition effect if applied during conditioning, and eliminating latent inhibition if applied during testing [43] (Fig 5B). The link between the salience signal (*S*(*t*), determined by levels of excitatory unit activity) and Amygdala activity can be better understood by examining how each changed from one trial to the next (Fig 5C and 5D). As illustrated by the downward slope of activity among Cortex excitatory units over pre-exposure trials, the network receiving pre-exposures learned to treat the CS as irrelevant (Fig 5C). As a result, during the conditioning period (gray shaded area in Fig 5C and 5D), Cortex activity was too low to push the Amygdala past threshold, making it less likely for learning to occur in the Amygdala in the pre-exposure condition (gray dotted line in Fig 5C and 5D). In contrast, the excitatory activity during conditioning for the non pre-exposure condition was high due to relevance learning, which resulted in a sufficiently strong Amygdala response to the CS to induce fear association learning (gray solid line in Fig 5C and 5D). When inhibitory signaling was experimentally reduced during conditioning (red lines in Fig 5C and 5D), both Cortex activity and Amygdala learning were amplified. However, this learning led to greater than normal inhibition during the test phase, so that in the pre-exposure condition the network remained relatively inactive during testing (red dotted line in Fig 5C and 5D; compare to control pre-exposure condition, grey dotted line). In contrast, when inhibitory signaling was reduced during testing, many Cortex units become active in both the pre-exposure and no pre-exposure conditions (blue lines in Fig 5C). The key to our competitive learning algorithm, though, is that the Amygdala weights align to the excitatory inputs (Fig 4). Thus, the over-activity of the Cortex units actually made it slightly harder for the Amygdala to pass threshold during testing (blue lines in Fig 5D). Hence, our model qualitatively recapitulated the results of Piantadosi & Floresco (2014) thanks to the interaction between relevance learning and the threshold effects in our Amygdala output layer. These data provide a new interpretation of the Piantadosi & Floresco (2014) experiments. Specifically, they suggest that by manipulating the inhibition in cortex, Piantadosi & Floresco (2014) may have been altering the encoding of stimulus relevance, and thereby, affecting the behavior of a downstream circuit, such as the amygdala, that may respond/learn from relevant stimuli using a threshold mechanism.

### Inhibitory relevance-learning network with amygdala module recapitulates effect of optogenetic manipulations on fear behavior

**Fig 6:**
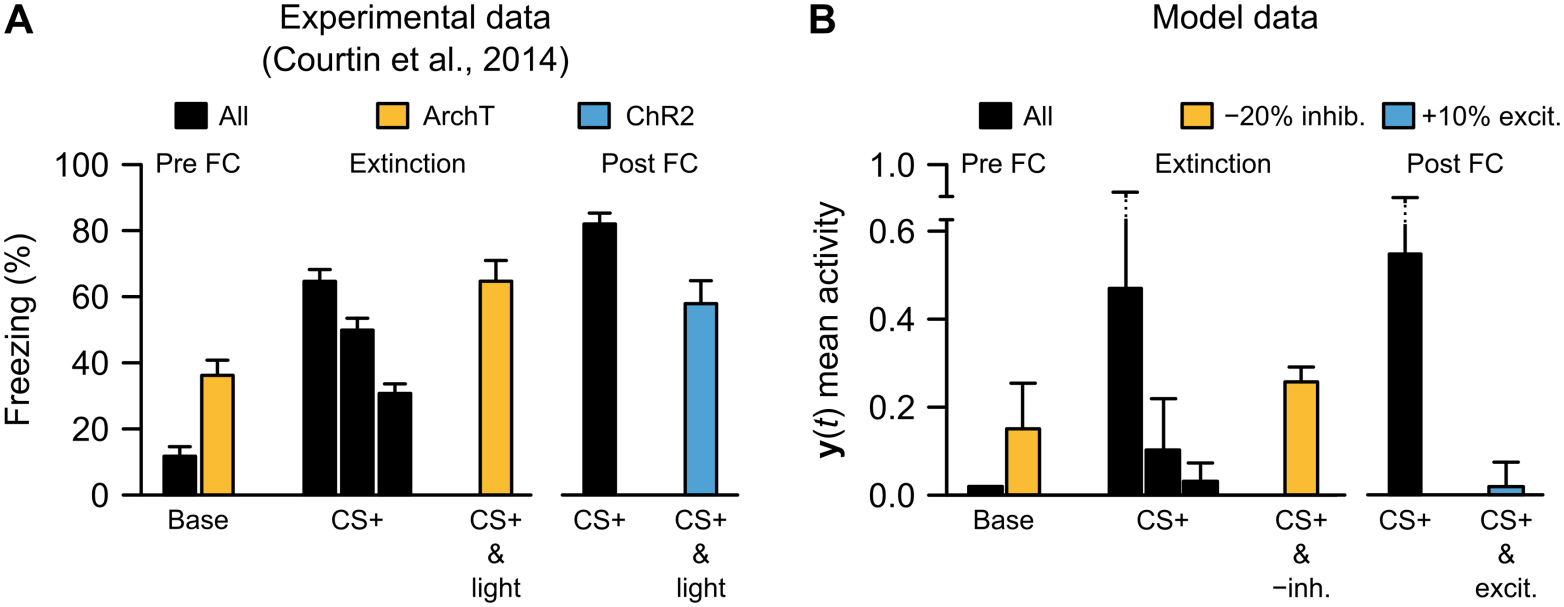
Simulation of experimental data on role of feedforward inhibition in freezing behavior. (**A**) Experimental data illustrating the effects of optogenetic inhibition (“ArchT”) or excitation (“ChR2”) of medial prefrontal cortex PV+ inhibitory neurons (reproduced by hand from [44]). Inhibitory neuron inhibition was performed both before conditioning (“Base”) and following conditioning and extinction (CS+ & light). As well, inhibitory neuron excitation was performed following conditioning (right side, CS+ & light). (**B**) Replication of general patterns of inhbitory neuron manipulations in the model, substituting -20% inhibition for “ArchT” and +10% excitation (i.e., increased ***W***^**I→E**^ weights by 10%) for “ChR2”.

The second simulation of experimental results we conducted addressed work by Courtin et al. (2014) [44], which examined how the activity of PV+ interneurons in the mPFC controls fear expression. As mentioned above, PV+ interneurons are the cells that we intended to model using the Cortex inhibitory unit, *I*(*t*). We simulated the experiments of Courtin et al. (2014) using the same network and parameters as used to simulate latent inhibition above (see Methods). The original study by Courtin et al. (2014) demonstrated that optogenetic stimulation of PV+ interneurons in the mPFC results in increased fear responses in mice, both before conditioning and, even more prominently, when stimulation was paired with a CS following extinction [44] (Fig 6A, *left*). When we applied the same protocol to our model, using a reduction in inhibitory inputs to mimic optogenetic silencing (see Methods), the same pattern of activity was observed in the Amygdala layer (Fig 6B, *left*). The original experiments also showed that activation of mPFC PV+ interneurons decreased freezing to a conditioned CS (Fig 6A, *right*). This was consistent with activity patterns in the model in a subsequent set of simulations (Fig 6B, *right*). As with the latent inhibition tests above, our results here provide a novel interpretation for the Courtin et al. (2014) study. Specifically, our data suggest that the effects of silencing or activating PV+ inhibitory interneurons in the mPFC may be explained by the interaction between a relevance code mediated by feedforward, divisive inhibition, and a threshold mechanism in the amygdala. They also offer evidence that the present model, in spite of its simplicity, may capture an essential relationship between the role of inhibition in the mPFC region and the competitive network in the amygdala [65, 66].

### Relevance learning can be multiplexed with input classification

A final set of simulations was used to investigate a key computational advantage to using the overall level of excitation for signaling relevance. If the overall level of excitatory activity encodes relevance (via *S*(*t*)), and this is controlled by feedforward inhibition, then the excitatory synapses in the network should still be free to control the specific pattern of **E**(*t*) to encode other information. This can be described mathematically by viewing the excitatory Cortex activity patterns **E**(*t*) as vectors, where the norm (length) of the vector is a signal of relevance (*S*(*t*)), but the position that the vector points in encodes other aspects of a stimulus, such as orientation, frequency, or category.

**Fig 7:**
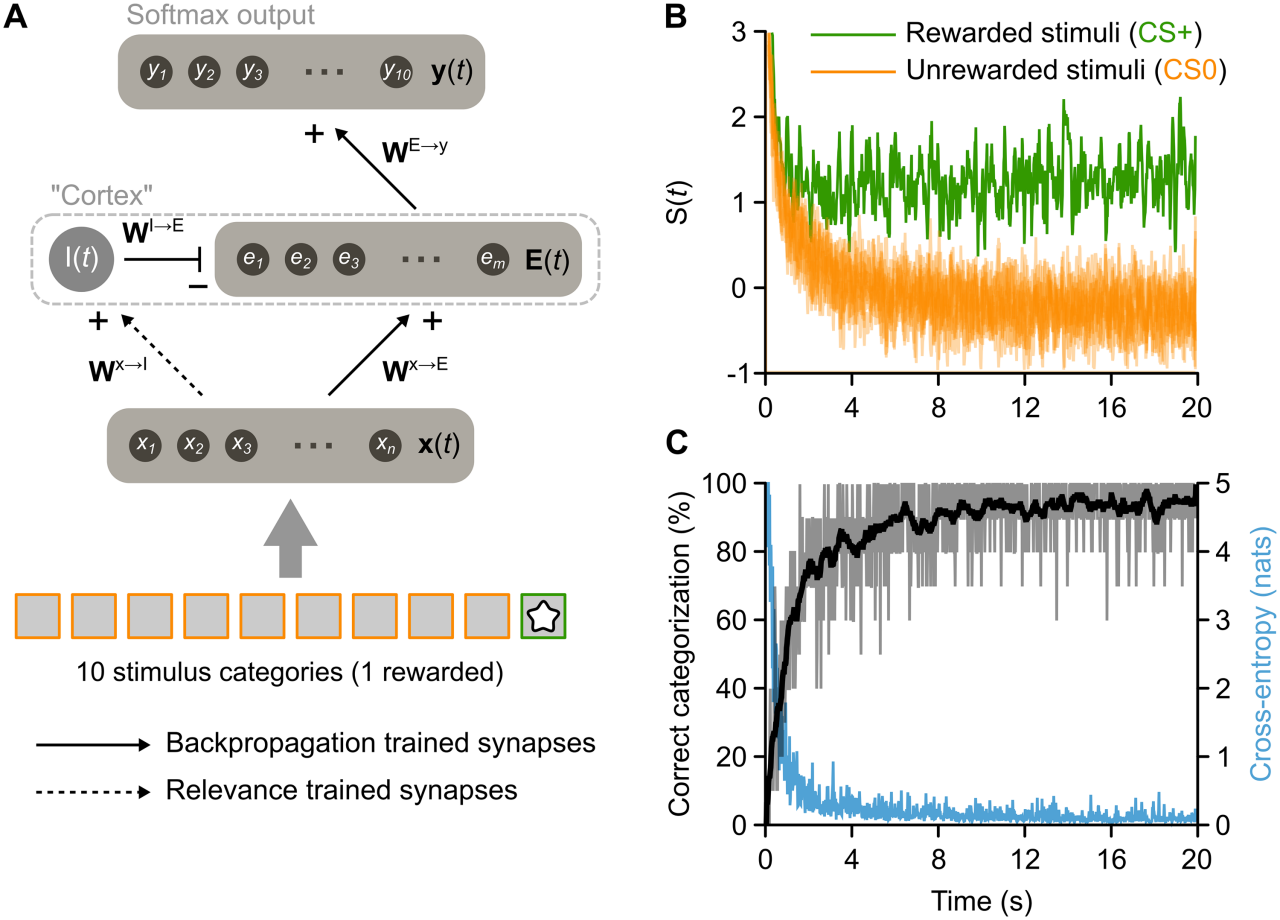
Multiplexed stimulus category and relevance codes via simultaneous excitatory and inhibitory learning. (**A**) Diagram illustrating modified model that included both the mechanisms described above for relevance learning (on ***W***^**x→I**^ synapses) in addition to mechanisms learning an output vector that matches categories presented as input (backpropagation algorithm applied to the ***W***^**E→y**^ and ***W***^**x→E**^ synapses). As illustrated by bottom boxes, one of ten stimuli presented to the network was rewarded. (**B**) Average excitatory unit responses to the one rewarded (green) and nine unrewarded stimuli (orange) over time. The network quickly learns to respond more strongly to the rewarded stimuli. (**C**) Performance of the model on input classification. Over the same time that the network learns to discriminate rewarded and unrewarded stimuli, it also becomes capable of matching the output vector to the input. The gray trace shows the percent of presentations that stimuli are correctly classified, which increases quickly before reaching a plateau. The blue trace shows the cross-entropy, an information measure (in natural units of information) based on the output activity distribution that is inversely related to the success of input classification.

To test this idea, the network was trained to categorize 10 different stimulus classes, with only one of these paired with a reward. The prediction was that the network could learn information about relevance and also learn to respond with output patterns specific to the correct stimulus category. To train the network to categorize stimuli, we employed a softmax Output layer (see Methods) and trained the excitatory pathway in the network with backpropagation-of-error [67] (Fig 7A). It is worth noting that although backpropagation-of-error is not a biologically realistic learning algorithm, there is evidence that it could be approximated with biologically realistic mechanisms [68, 69, 70, 71]. Furthermore, independent of the specific algorithm used, the goal of the simulation was simply to offer a proof of the multiplexing concept.

Training of the excitatory pathway with backpropagation was done concurrently with training of the feedforward inhibition pathway using the relevance learning algorithm (as described in Methods). Over the course of training, the network learned to dissociate the rewarded stimulus category from the unrewarded ones, via the relevance signal, *S*(*t*) (Fig 7B). Importantly, *S*(*t*) did not differentiate between the unrewarded categories (Fig 7B, *orange lines*), demonstrating that it was not encoding the categories, *per se*, but only their relevance for predicting reward. At the same time, the set of ‘Category’ output units did learn to differentiate all 10 categories of stimuli. A cross-entropy loss function was used to evaluate the success of categorization, with lower values indicating a higher degree of separation between the categories. Over the course of 20 simulated seconds of training, this measure dropped to almost zero and the output layer was achieving roughly 95% accuracy on average (Fig 7C). We found similar results when we rewarded three of the stimuli, rather than only one (S4 Fig). Importantly, relevance learning and category learning were operating simultaneously in these simulations. The results demonstrate the potential for multiplexing relevance signals with other stimulus information by using the overall level of excitatory activity as a code for value.

## Discussion

The simulations presented here explored how disruptions in feed-forward, neural inhibition could compromise the brain’s ability to ignore irrelevant inputs, as observed in schizophrenia. The model was structured as simply as was necessary to examine this connection, incorporating three core mechanisms. First, relevance was coded as the overall excitatory activity in the ‘Cortex’ layer. Specifically, the norm (length) of the excitatory units’ activity vector was treated as a reinforcement learning value function, though “unsigned” in that it treated positive (reward) and negative (punishment) values equivalently (Fig 1). Second, the model used feedforward inhibition—i.e., the connections from the ‘Sensory’ input layer to Cortex inhibitory units—to control the overall level of Cortex excitatory activity. When paired with the first mechanism, the result was that disruptions to inhibition led to failures in normal relevance attribution (Figs 2 & 3). Third, the model used a form of an established reinforcement learning algorithm, temporal difference learning, to train the feedforward inhibitory connections and thereby learn to differentiate relevant versus irrelevant stimuli. When these mechanisms were further connected in sequence with an output (the ‘Amygdala’) that used a threshold and a competitive learning mechanism (Fig 4), they offered specific predictions about how disruptions to inhibition alter fear behavior (Figs 5 and 6).

These three mechanisms are highly consistent with previous empirical work. The idea that overall levels of excitation in “Cortex” may provide a code for unsigned value was inspired by work on the medial prefrontal cortex (mPFC), a region that has been implicated in schizophrenia and many other disorders [72]. Recent data has demonstrated the importance of mPFC disinhibition for coding relevant situations [33, 44, 73, 74, 75], including the observation that net levels of activity in putative pyramidal neurons increase near reward sites [38]. The second mechanism, assigning control of this relevance code to feedforward inhibition, matches empirical findings on the importance of inhibition for behaviors like latent inhibition (e.g. [43]). It also matches decades of work linking relevance impairments in schizophrenia [1, 2, 3, 4, 5, 8, 9] with evidence that inhibitory neurons, and in particular, classes of inhibitory neurons supporting feedforward inhibition, may be differentially compromised in the disease [14, 15, 16, 17, 18, 76]. Finally, the third mechanism, wherein inhibitory interneuron plasticity is the means for learning to differentiate relevant versus irrelevant stimuli, is consistent with findings that the neural connections supporting feedforward inhibition are plastic [77, 78, 79], in some cases requiring NMDA receptors with a well established importance for associative plasticity [80, 81, 82]. These three mechanisms together comprise a more general theory of cortical coding: plasticity involving inhibitory neurons may act in parallel with excitatory neuron plasticity to accomplish different learning functions. While excitatory plasticity may provide a mechanism for carrying information about stimulus specifics, plasticity involving inhibitory neurons may be important for relevance learning (Fig 7). This suggests a multiplexing of learning functions in the neocortex, and links a large literature on inhibitory plasticity with theories about the importance of these neurons for maintaining EI balance.

As this was an abstract neural network model, many features of the real brain were absent. The most notable was the absence of feedback connections within Cortex. By excluding these connections, the mathematical complexity of calculating synaptic balances and their experience-dependent changes could be minimized, and it became possible to isolate the learning algorithms that explain the behavioral phenomena of interest. The results demonstrate that plasticity in the synapses connecting inputs to inhibitory neurons is sufficient to support relevance learning. Such a mechanism also causes relevance learning to be dysfunctional following disrupted inhibition. In contrast with our model, which lacks feedback excitatory connections, Murray and colleagues used a more detailed circuit model that included these connections to show how inhibition helps maintain intact memory representations, and how this could be disrupted in schizophrenia [21]. The aim of this previous study was very different from the present investigation; the findings, however, are not inconsistent. It would be beneficial in the future to examine the interrelationships between functions and algorithms of feedforward versus feedback excitation, including the dependencies that may exist between working memory and stimulus gating.

Another feature missing from the present model was the absence of different types of inhibitory neurons. Recent work by Yang et al. [83] tackled the question of how the inhibitory system regulates signal propagation (“gating”) using functionally distinct types of inhibitory neurons. They were able to show how signal propagation may require parallel signaling between disinhibitory and excitatory inputs onto the same neurons. This model highlights some key features that distinguish the present work from other research in the area ([20, 83]). Most obviously, our model does not adhere to the requirement that EI balance must be strictly maintained: regulation of signal propagation takes place at the population level by allowing for dynamic EI balance (discussed above). Relatedly, no signals are completely gated within the cortex: “relevant” versus “irrelevant” information is differentially represented with relatively subtle, average firing rate differences across the population of all neurons. It is only when Cortex signals reach an efferent region (in our case, the competitive-learning Amygdala) that information related to particular input patterns is prevented from propagating forward. Indeed, in our multiplexing experiments the category information of irrelevant stimuli was maintained (Fig 7). These two distinctions, the population-level regulation of signal propagation and the graded way in which it is implemented, provide the basis for multiplexing of learning functions, by allowing inhibitory and excitatory plasticity processes to follow independent learning rules (discussed below). The population-level approach is also intuitively consistent with the necessarily high-dimensionality of single-neuron coding in regions like prefrontal cortex (e.g., [84]). Moreover, recent computational work suggests that ensemble activity in cortical pyramidal neurons can itself multiplex feedforward and feedback signals [85]. Such a mechanism, paired with our results, could provide a means of simultaneously encoding relevance, stimulus identity, and top-down information (e.g. feedback or attention) in the same cortical microcircuits.

While the abstract nature of the model offers only a proof-of-concept for certain elements of true cortical computation, it also raises potentially fundamental questions about how certain processes may be implemented in the brain. Particularly compelling is the question of where the computed salience signal (*S*(*t*)) and corresponding prediction error (*β*(*t*)) come from. We consider two non-exclusive possibilities. In one scenario, the salience signal is explicitly read-out by cells in neuromodulatory nuclei, as has been described within the dopaminergic system [86], which is then used to compute a prediction error signal that feeds back to the cortical afferent, modifying local plasticity accordingly [87]. In the second scenario, the prediction error calculation responsible for maintaining EI balance is carried out by circuits that are local to the cortex, and may take place, for example, by intrinsic signaling processes within the inhibitory neurons (see also [88, 89]). In this case, either the salience signal itself (the excitatory input onto inhibitory neurons) or a set of intrinsic, cellular processes that compute the difference between inputs and “desired” output levels (the prediction error signals), are modified by neuromodulatory signals carrying information about current rewards/punishments.

However the prediction error signal is implemented, the plasticity processes involved must be intertwined with local mechanisms for maintaining EI balance; otherwise, EI balance maintenance would be constantly working to compensate for changes associated with relevance learning. As described in Introduction, the ability of local cortical circuits to maintain EI balance is well established [22, 23, 24, 25, 26, 27, 28, 29, 30, 31, 32, 90]. The process by which networks maintain this balance, while not fully known, has been proposed to be supported by plasticity at inhibitory synapses in response to feedback excitatory signals [88, 89]. Recent work has demonstrated local changes in synaptic scaling at both inhibitory and excitatory synapses following local changes in excitation [91]. One possibility is that while the local network is capable of maintaining EI balance, its set-point can be adjusted by signals from extrinsic neuromodulators. Dopamine has received much attention in signaling salience, but acetylcholine has also come under the spotlight (e.g. [36, 92, 93, 94]). Disinhibition may also involve a class of inhibitory neurons that contain vasoactive intestinal polypeptide (VIP+ interneurons [33]). To accurately capture the mechanisms of the supervisory process, it will likely be necessary to increase the complexity of the model by including feedback connections between excitatory neurons, and connections from excitatory to inhibitory neurons.

One detail that was critical in the present model, and a primary prediction of the present work, is that relevance learning not only involves inhibitory changes, but specifically may involve plasticity on the feedforward, input-to-inhibitory neuron synapses in cortical circuits. This prediction is consistent with recent data showing that the number of excitatory synapses onto parvalbumin-expressing inhibitory neurons is reduced in schizophrenia [95]. It also adds to a growing literature on the functions of inhibitory neuron plasticity [96, 97, 98]. One recently proposed idea is that during memory encoding, patterns of inhibitory modifications mirror excitatory modifications. This would ensure that EI balance can be appropriately maintained and thereby reduce inappropriate recall [96]. The present model fits with this proposal. Inhibitory synapses in the model learned to match any increases in excitatory synapses in order to keep the “Cortex” output at the homeostatic set-point. Only when the prediction error signal indicated that an input was relevant was this mirrored inhibition relaxed to allow the excitatory activity to increase.

The simulations also addressed how the proposed code for relevance might impact learning and activity in a post-synaptic region. With the aim of simulating experimental fear-learning data, the post-synaptic region chosen was the amygdala, which was modeled as a competitive learning network [64]. Use of a competitive network is consistent with known properties of the mammalian amygdala [65, 99]. Additionally, projections to the amygdala can be found throughout the mPFC (e.g. [100]) and these projections are known to excite principle neurons in that region (e.g. [101]). By combining our proposed relevance code at one layer of the model with a competitive learning rule with a threshold at the next, the model was capable of responding selectively to only those specific stimuli that had been paired with reinforcement signals in the past (Fig 4). The model thereby became capable of replicating behavioral patterns from both pharmacological [43] and optogenetic [44] manipulations to mPFC inhibitory neurons (Figs 5 and 6).

Our model shows how changes in inhibitory gain control can determine how excitatory activity is involved in representing distinct stimuli and, in the Amygdala simulations, how this can drive behavior and learning. It is important to emphasize that this idea in itself is not novel. Another notable model in which plasticity is modulated by stimulus-regulated gain control has been described by Harris and Livesey [102, 103]. This particular example uses a very different structure from our own network, and is aimed at a very different question: how associative learning can take place for stimulus combinations, even when the representations of such stimuli are “elemental” (see also [104]). It is also capable of replicating many classical conditioning phenomena that our own simulations do not address, and further work would be required to identify which features of the two models are compatible. What is perhaps most interesting, however, is not so much the differences between models, but the apparent utility and versatility of gain control regulation for network computation. The present work specifically focuses on how inhibitory plasticity may support some of these functions.

One novel contribution of the model is the mechanism it proposes for the multiplexing of different learning functions in cortical networks. By allowing inhibitory plasticity to rely on an entirely independent learning rule from excitatory neuron plasticity, we allow the network to perform associative learning on both relevant and irrelevant input patterns (Fig 7). The ability of the network to learn even in the absence of novelty or relevance could be thought of as a kind of implicit learning, in which knowledge of the environment—including its statistical structure—is extracted from experience in the absence of reinforcement, attention or consciousness [105, 106]. This differs from many other models, including those cited above, in which inhibitory plasticity is closely tied to excitatory plasticity (e.g., [83]). In the future it will be useful to examine the specific roles of inhibitory neuron plasticity in more detail, and to see whether the differences in approaches may be reconciled through different inhibitory neuron types, cortical layers, or other factors.

There are a number of behavioral phenomena, well reported in the classical conditioning literature, that fall outside of the scope of our simulations. One that has been observed since some of the earliest experiments by Pavlov and Konorski is the phenomenon where a stimulus not associated with an US can become a “conditioned inhibitor”—i.e., it can become salient in its own right, inhibiting responses normally associated with the US [107]. An example of this is if the pre-exposed CS in the latent inhibition paradigm comes to be perceived as a salient “safety cue”. The extent to which behaviors like latent inhibition are determined by the CS becoming a conditioned inhibitor is unclear, and likely depends on the specific protocol used. An interesting possibility is that different cortical areas make use of a similar scalar code for relevance, but apply them to different—and sometimes opposing—functions. Within the mPFC the infralimbic cortex seems to be involved in signaling safety, important for fear extinction, while the slightly more dorsal, ventral prelimbic cortex may instead signal danger, important for fear learning (e.g., [108, 109]). If a punisher is assumed by default given past history and context, then the absence of the punisher may effectively act as an US, suppressing inhibitory neuron activity. In the mPFC this may engage the infralimbic cortex to promote the signaling and learning of safety. If a punisher is not assumed, then, according to our framework, the only effect of its absence following a CS0 would be plasticity processes on inhibitory neurons to maintain excitatory-inhibitory balance, resulting in loss of attention to the CS0 and retardation of learning a subsequent CS-US pairing (see S5 Fig). Explorations of the differences between learning the relevance of a stimulus for safety, punishment, or reward in different circuits were outside of the scope of the current study, but should be explored in future work.

Another area that was not explored in the current set of experiments was the increasingly apparent link between pathologies of EI balance and deficits in social behavior and motivation [110, 111, 112]. It seems likely, however, that some of the more basic results from the present investigations could offer understanding for why some individuals have more difficulty filtering or dynamically processing social information. Tackling these complex problems will require a convergence of multiple experimental and theoretical approaches, and mathematically tractable network models that include excitatory-inhibitory interactions will be an essential tool.

Altogether, our theoretical investigations provide a potential explanation for why behaviors such as gating and relevance learning could depend on feedforward inhibition, and therefore, how pathologies of inhibition may underlie neuropsychiatric conditions such as schizophrenia. In many ways the ideas reformulate a long existing hypothesis that schizophrenia is a disruption of feedforward inhibition [9]. But the model offers a computational description of the process with defined links between several functional elements. Furthermore, it offers valuable predictions about the importance of plasticity in both excitatory and inhibitory neurons, lending insights into the normally functioning brain.

## Methods

### Network summary

The core of the model is a two-layer feedforward neural network composed of different types of units. Stimuli are encoded by a set of input units, **x**(*t*) = [*x*_1_(*t*), …, *x*_*n*_(*t*)], the ‘Sensory’ layer. In our analysis of the network, we treat **x**(*t*) as a vector of firing rates. In our simulations, this layer is a set of excitatory Poisson units with firing rates $\phi^{x}\left(t\right)=\left[\phi_{1}^{x}\left(t\right),\ldots ,\phi_{n}^{x}\left(t\right)\right]$. Changes in the Sensory layer take place when stimuli are presented, as described in more detail below. Sensory units feed into a middle layer, ‘Cortex’, that is comprised of two populations: an excitatory population, **E**(*t*) = [*e*_1_(*t*), …, *e*_*m*_(*t*)], and an inhibitory population modeled as a single unit *I*(*t*) that acts divisively on the excitatory units. As with the sensory inputs, we treat **E**(*t*) as a vector of firing-rates when conducting our analyses, but simulate it as a vector of Poisson units with rates $\phi^{E}\left(t\right)=\left[\phi_{1}^{E}\left(t\right),\ldots ,\phi_{m}^{E}\left(t\right)\right]$. The receptive fields of Cortex units have no temporal dimension, so the activity at any point only reflects the current inputs to the network.

The connections from input units to the excitatory cortex units are contained in an *m* × *n* synaptic weight matrix, **W**^*x*→*E*^, the connections from input units to the inhibitory Cortex unit are contained in the *n*-dimensional vector of synaptic weights, **W**^*x*→*I*^, and the connections from the inhibitory unit to the excitatory units are contained in an *m*-dimensional vector of synaptic weights, **W**^*I*→*E*^.

Altogether, this set-up gives the following equations which describe the activity of the model in the simulations:

$$\begin{array}{c} \begin{array}{cc} x(t) & ∼Poisson(dt\phi^{x}\left(t\right))\\ I(t) & =W^{x\to I}\cdot x\left(t\right)+b^{I}\\ \phi^{E}\left(t\right) & =\left(W^{x\to E}\cdot x\left(t\right)+b^{E}\right)⊘\left(W^{I\to E}I\left(t\right)+I_{floor}\right)\\ E(t) & ∼Poisson(dt\phi^{E}\left(t\right)) \end{array} \end{array}$$ (12)

where ⊘ represents element-wise division of a vector/matrix, *dt* is the time-step, which is 20 ms for most simulations, ***b***^**E**^ and *b*^**I**^ are bias terms, and *I*_*floor*_ = 0.1 prevents division by zero. We note that here we have indicated *I*(*t*) as a scalar, since it was in most simulations, but it can be formulated as a vector with no change to the results (see S3 Fig). As well, we note that for our mathematical analyses and gradient calculations we simply set **x**(*t*) = *ϕ*^*x*^(*t*) and **E**(*t*) = *ϕ*^*E*^(*t*).

One additional component that is not included in the above equations, but which contributes to relevance learning (see Relevance Learning, below), is a signal communicating the unsigned magnitude of reward or punishment, i.e. the unconditioned stimulus (US). In the present simulations the value of the US at a given time (*u*(*t*)) is either 1 or 0, though in principle it could as easily be a graded value.

In some simulations, we add an additional output layer of units with activity **y**(*t*) = [*y*_1_(*t*), …, *y*_*ℓ*_(*t*)] that receives inputs from the excitatory cortical units via an *ℓ* × *m* synaptic weight matrix, **W**^*E*→*y*^. In those simulations which address previous experimental findings (Figs 5 and 6), the output layer is intended to represent an amygdala (‘Amygdala’) and implements a competitive learning algorithm (according to the framework of [64]). In the competitive learning module, a maximum of only one unit may be active at any given time (it is possible for no units to be active). Whether a unit, *i*, is active depends on two conditions: (1) the unit is receiving stronger input than any of the other units, (2) the unit’s input, $W_{i}^{E\to y}\cdot E\left(t\right)$, is greater than a threshold, *θ*. Amygdala units also receive signals from the US, such that *u*(*t*) can help to increase output, *y*_*i*_(*t*). Based on all of this, the activities of the Amygdala units are governed by the following equations:

$$\begin{array}{c} \begin{array}{cc} z_{i}\left(t\right) & =\sum_{j}W_{ij}^{E\to y}e_{j}\left(t\right)-\theta \\ y_{i}\left(t\right) & =\{\begin{array}{cc} z_{i}\left(t\right)+0.5u\left(t\right) & \text{if}\;z_{i}\left(t\right)>z_{j}\left(t\right)\text{,}\;\forall j\neq i\;\text{and}\;\left(z_{i}\left(t\right)\geq 0\;\text{or}\;u(t)>0\right)\\ 0 & \text{otherwise} \end{array} \end{array} \end{array}$$ (13)

Note that the activities *y*_*i*_(*t*) are rescaled after every weight update (see below) such that $y_{i}\left(t\right)\leftarrow \frac{y_{i}\left(t\right)}{\sum_{i}z_{i}\left(t\right)}$ and $\sum_{j}W_{ij}^{E\to y}=1$. This rescaling provided an important normalization of the Amygdala activity, keeping it in a reasonable range without impacting learning.

The threshold, *θ*, determines when the Amygdala layer can have any active units. An explanation for how *θ* was selected is given in the results in ‘Effect of relevance learning on downstream circuitry’.

Although having a single neuron firing is undoubtedly not what occurs in the mammalian amygdala, there is evidence for a competitive “winner-takes-all” mechanism [65, 66], such that a single ensemble of neurons is active and all others are silent. Therefore, the active unit in our model ‘Amygdala’ could be taken to represent an ensemble of “winning” neurons. Since individual units in this case were representative of larger ensembles, the winning unit’s firing rate was kept as a continuous “activation level” value rather than Poisson-distributed spike counts.

The output layer takes on a different form in those simulations that demonstrate how our model can multiplex relevance signals and stimulus identity. In this case, the output units represent some efferent, such as a second area of cortex, that is responsible for categorizing input activity. For simplicity, we refer to this layer in the simulations as the ‘Category’ layer. The Category layer is a set of softmax, linear-non-linear-Poisson units with rates *ϕ*^*y*^(*t*) governed by:

$$\begin{array}{c} \begin{array}{cc} \phi_{i}^{y}\left(t\right) & =\kappa \frac{\sum_{j}W_{ij}^{E\to y}e_{j}\left(t\right)}{\sum_{k}\sum_{j}W_{kj}^{E\to y}e_{j}\left(t\right)}\\ y_{i}\left(t\right) & ∼Poisson(dt\phi_{i}^{y}\left(t\right)) \end{array} \end{array}$$ (14)

where *κ* = 20 Hz scales the firing-rates such that the activity of the units is proportional to the probability of each of the *ℓ* possible categories for the current stimulus, with a rate-of-fire of 20 Hz corresponds to a probability of 1.

### Stimuli

Sensory (input) units are divided into sets of stimulus-coding and non-coding units. Each stimulus is capable of activating one tenth of the Sensory units. In the case of the learning to ignore simulations, for example (see Learning to ignore and blocking, below), one tenth of the units are activated by the stimulus, “CS+” that is paired in time with the US, one tenth of the units are activated by another stimulus, “CS0”, that are random in time relative to the US, and the rest are activated by neither CS+ nor CS0. Importantly, Sensory units fire both when activated by a stimulus and not, just at different rates. An active sensory unit generates spikes with a Poisson process at a rate of *ϕ*^*on*^ = 20 Hz, while an inactive sensory unit generates spikes with a Poisson process at a rate selected randomly based on a gamma distribution that peaks at 0.6 Hz and has a variance of 3 Hz^2^. These rates were selected based on baseline firing characteristics among putative excitatory neurons recorded from the rat medial prefrontal cortex [38]. Hence, for example, if the CS+ is presented to the network, then the ten percent of the units activated by CS+ will be firing at a rate of 20 Hz, while other units will continue firing at their typically low (0-2 Hz) but occasionally high (10 or 20 Hz) baseline rate. In those simulations that use the Category output layer, Sensory units are divided into ten sets, with each set activated differentially by a particular category (in these simulations, baseline rates were also simplified to be homogeneously 2 Hz, as variance was found to not impact the results).

### Weight initialization

Initialization of the three sets of connection weights in the first layers—Sensory to Cortex excitatory units (**W**^*x*→*E*^), Sensory to Cortex inhibitory unit (**W**^*x*→*I*^), and Cortex inhibitory to excitatory units (**W**^*I*→*E*^)—took into account three issues. First, when novel stimuli were first presented to the network, the evoked activity in Cortex excitatory units needed to be higher than baseline levels, but ideally not much higher than levels associated with “relevant” stimuli (described below in Relevance learning). Second, baseline activity of the cortex inhibitory unit had to be high enough that reducing **W**^*x*→*I*^ had an impact on Cortex excitatory units. Third, that **W**^*x*→*I*^ were balanced with **W**^*x*→*E*^, such that small changes in **W**^*x*→*I*^ could not dramatically alter population activity. These three constraints were additionally considered alongside the targeted, average firing rates associated with “relevant” and “irrelevant” input vectors. Based on data from Insel and Barnes [38], these corresponded to average firing rates in regular-firing, wide-waveform neurons of 3.9 (at reward sites) and 2.6 Hz (during quiet waking) respectively (see also Relevance Learning, below). With these constraints and target firing rates, a grid search was used to search for a combination of 4 parameters to set the starting weights of the network: 1) fixed starting weights for **W**^*x*→*I*^ (*a*^*x*→*I*^), 2) fixed starting weights for **W**^*I*→*E*^ (*a*^*I*→*E*^), 3) center of Gaussian for **W**^*x*→*E*^ (*μ*^*x*→*E*^), 4) variance of the Gaussian for **W**^*x*→*E*^ (*σ*^*x*→*E*^). Once the target firing-rates had been met by the weight parameters, the grid search was ended. This produced values of *a*^*x*→*I*^ = 0.5, *a*^*I*→*E*^ = 0.4, *μ*^*x*→*E*^ = 0.3, and *σ*^*x*→*E*^ = 0.4. It is important to note that this initialization grid search did not make learning any easier, because nothing about the initialization contained stimulus information. All that the initialization search did was provide physiologically realistic firing rates. It would likely be possible to satisfy the same constraints and firing rate patterns using different initialization parameter sets, but this was not explored.

To summarize, starting weights for the Cortex for most simulations were set as follows: $W_{i}^{x\to I}=0.5\forall i$, $W_{i}^{I\to E}=0.4\forall i$, and $W_{ij}^{x\to E}∼N\left(\mu^{x\to E},\sigma^{x\to E}\right)\forall i,j$. The one exception to this is the latent inhibition simulations shown in (Fig 5). In these simulations we maintained the stimulus information in the initial condition to a greater degree by setting the weights **W**^*x*→*E*^ to be a smoothed diagonal matrix.

All weights from the Cortex to the output units were initialized using a uniform random distribution: $W_{ij}^{E\to y}∼U\left(0,1\right)$.

Finally, we note also that in all our simulations we respected “Dale’s” law by clipping any negative connections weights at zero. (Clipping at zero did not prevent later increases to the weights). This was done both for initialization and during learning. All of the specific implementations of these initialization procedures can be found by downloading our code (see the repository link below).

### Relevance learning

The principal learning mechanism used in this paper is a modification of the temporal difference learning algorithm [39]. Specifically, a population-based relevance (or “salience” signal), *S*(*t*), reflects the deviations in excitatory activity from an established baseline. The baseline level can be thought of as the EI balance set-point maintained by the cortical network. The level of Cortex excitatory unit activity was measured as the vector norm of the population of excitatory units, ${∥E\left(t\right)∥}_{2}=\sqrt{\sum_{i}e_{i}{\left(t\right)}^{2}}$ (the reasons for using the norm become clear in Eq 10). *S*(*t*) is therefore determined by the difference between ∥**E**(*t*)∥_2_ and the homeostatic set-point for the population, *H* (Fig 1B):

$$\begin{array}{c} S\left(t\right)={∥E\left(t\right)∥}_{2}-H \end{array}$$ (15)

(Note: this equation is identical to Eq 1 in the Results). The goal of relevance learning in the network is to have *S*(*t*) come to represent expected relevance, which we interpret as “unsigned value”, *U*(*t*):

$$\begin{array}{c} U\left(t\right)=\langle \sum_{i=1}^{\infty }\gamma^{i-1}u\left(t+i\right)\rangle \end{array}$$ (16)

where *u*(*t*) is the unsigned reward/punishment signal, US, described above, *γ* is a temporal discounting factor, and 〈⋅〉 indicates expected value. *U*(*t*) is akin to the value function used in temporal difference learning [39]. Similar to temporal difference learning, the goal of learning in our model is, in part, to ensure that *S*(*t*) is a good estimate of *U*(*t*). This is accomplished using a prediction error signal, *β*(*t*):

$$\begin{array}{c} \beta (t)=Au(t)+\gamma S(t)-S(t-1) \end{array}$$ (17)

where *A* is a salience scaling factor that determines how much cortical activity levels should deviate from the set point in response to relevant stimuli. We use *β* to represent our prediction error signal, rather than the usual *δ*, to distinguish it from prediction error signals that measure differences in signed (as opposed to unsigned) value estimates [39]. (The notation also deviates slightly from convention by using *t* rather than *t* + 1, to avoid questions about whether the model has future information. This is just a re-indexing, though, and does not affect the results in any meaningful way).

To put it another way, the system learns to ensure that fluctuations in ∥**E**(*t*)∥_2_ away from the set-point, *H*, reflect experience with rewards/punishments (the US). The scale of the fluctuations is determined by *A*. Training the salience signal *S*(*t*) involves updating the synaptic weights in Cortex to achieve *β*(*t*) = 0. It can be seen that this is achieved when:

$$\begin{array}{c} {∥E\left(t\right)∥}_{2}=H+AU\left(t\right)\Rightarrow S\left(t\right)=AU\left(t\right) \end{array}$$ (18)

since *U*(*t* − 1) = 〈*u*(*t*)〉 + *γU*(*t*). Therefore, *β* is generally close to zero when the following conditions have been achieved: (1) for stimuli that do not predict any reward or punishment, the norm of the spike count in the excitatory cortical population is equal to the homeostatic constant, *H* and (2) for stimuli that do predict reward or punishment *S*(*t*) is a linear function of *U*(*t*) with a slope of *A*.

To learn this, we perform stochastic gradient descent on the squared difference between *S*(*t*) and *AU*(*t*) (see (3)). If we treat **x**(*t*) and **E**(*t*) as rates of fire, it can be shown that:

$$\frac{\partial {\left(S\left(t\right)-AU\left(t\right)\right)}^{2}}{\partial W_{j}^{x\to I}}=\frac{\beta (t)}{{∥E\left(t\right)∥}_{2}}[{\left(W^{I\to E}\right)}^{T}\cdot (\left(E\circ E\right)⊘(W^{I\to E}I\left(t\right)))]x_{j}\left(t\right)$$ (19)

where ∘ indicates element-wise multiplication. Because we followed Dale’s law in our simulations, and firing rates can only be positive, none of the terms in Eq 19 can be negative except for *β*(*t*). Moreover, the only element of the equation that helps to differentiate Sensory inputs is *x*_*j*_(*t*). Thus, all of the other terms in Eq 19 can be treated as scaling terms. What this means is that the gradient direction in weight space is specified solely by *β*(*t*) and *x*_*j*_(*t*), while the other terms merely indicate the magnitude of the gradient in these directions. In practice, gradient descent can still occur when following the gradient direction, even if the magnitude of the gradient is ignored. Thus, this allowed us to simplify this expression and use only *β*(*t*) and *x*_*j*_(*t*) as in Eq 4, while still achieving the same results as would be obtained from following the true gradient defined by Eq 19.

In some simulations (Figs 2 and 3), the performance of this learning rule is compared against rules in which we perform gradient descent on either the **W**^*x*→*E*^ or **W**^*I*→*E*^ synapses. The partial derivatives for the squared difference between *S*(*t*) and *AU*(*t*) with respect to these weights are:

$$\begin{array}{c} \begin{array}{cc} \frac{\partial {\left(S\left(t\right)-AU\left(t\right)\right)}^{2}}{\partial W_{ij}^{x\to E}} & =-\frac{\beta (t)}{{∥E\left(t\right)∥}_{2}}e_{i}\left(t\right)x_{j}\left(t\right)\\ \frac{\partial {\left(S\left(t\right)-AU\left(t\right)\right)}^{2}}{\partial W_{i}^{I\to E}} & =\frac{\beta (t)}{{∥E\left(t\right)∥}_{2}}[\frac{e_{i}{\left(t\right)}^{2}}{W_{i}^{I\to E}I\left(t\right)}]I\left(t\right) \end{array} \end{array}$$ (20)

which we can simplify again thanks to Dale’s law and positive firing rates, giving us approximations of the gradients:

$$\begin{array}{c} \begin{array}{cc} \frac{\partial {\left(S\left(t\right)-AU\left(t\right)\right)}^{2}}{\partial W_{ij}^{x\to E}} & \propto -\beta \left(t\right)x_{j}\left(t\right)\\ \frac{\partial {\left(S\left(t\right)-AU\left(t\right)\right)}^{2}}{\partial W_{i}^{I\to E}} & \propto \beta (t)I(t) \end{array} \end{array}$$ (21)

which we then use for the weight updates:

$$\begin{array}{c} \begin{array}{cc} W_{ij}^{x\to E} & \leftarrow W_{ij}^{x\to E}+\alpha \Delta W_{ij}^{x\to E}\\ \Delta W_{ij}^{x\to E} & =-\frac{\partial {\left(S\left(t\right)-AU\left(t\right)\right)}^{2}}{\partial W_{ij}^{x\to E}}\\ W_{ij}^{x\to E} & \leftarrow W_{i}^{I\to E}+\alpha \Delta W_{i}^{I\to E}\\ \Delta W_{i}^{I\to E} & =-\frac{\partial {\left(S\left(t\right)-AU\left(t\right)\right)}^{2}}{\partial W_{i}^{I\to E}} \end{array} \end{array}$$ (22)

Using these weight updates for relevance learning can theoretically provide the same coding for relevance in *S*(*t*). However, they make different predictions regarding the effects of impaired inhibition (Figs 2 and 3).

As already noted in the previous section, the specific *H* and *A* used corresponded to empirical data measuring the average firing rates in the rat medial prefrontal cortex, with *H* = 6.5 Hz and *A* = 1.4 Hz, as observed by Insel and Barnes [38].

### Amygdala learning

In simulations with output units, **y**(*t*), such as the Amygdala, synapses between Cortex excitatory units and the Output units were also trained. In simulations using an Amygdala output layer, the Cortex-to-Amygdala weights, **W**^*E*→*y*^, were trained with a competitive learning algorithm as defined in Eq 8. As outlined in Effect of relevance learning on downstream circuitry, a suitable threshold, *θ*, can be found to ensure that in the *absence* of a US the Amygdala only responds to stimuli that have been paired with reward or punishment in the past. In the simulations presented here, the value of *θ* was set by grid search so that the probability of any neuron crossing threshold would be very low if no learning had occurred, and very high if an US was present or learning had converged and the network was presented with a relevant stimulus. The final value that was used in our simulations was *θ* = *H*/4.

### Categorization learning

In simulations where we trained the output units to categorize input stimuli, we used backpropagation-of-error [67] to train the weight matrices **W**^*x*→*E*^ and **W**^*E*→*y*^. More precisely, target vectors, **o**(**x**(*t*)) are defined, where each stimulus provided to Sensory units has a corresponding target vector for the output Category units. The cross-entropy [67] between the Category activity and target vectors was used as the loss function to train the network:

$$L(x(t))=\sum_{i=1}^{\ell }o_{i}\left(x\left(t\right)\right)\ln\left(y_{i}\left(t\right)\right)$$ (23)

where *o*_*i*_(**x**(*t*)) is the “target” response to input **x**(*t*) for output unit *i*, i.e. *o*_*i*_(**x**(*t*)) = 1 if *i* is the correct category for **x**(*t*), and it is zero otherwise.

For any weight *W*_*ij*_ in **W**^*x*→*E*^ or **W**^*E*→*y*^, the weight update is determined by the partial derivative of this loss function with respect to the weight:

$$\begin{array}{c} \Delta W_{ij}=-\alpha_{y}\frac{\partial L(x(t))}{\partial W_{ij}} \end{array}$$ (24)

where *α*_*y*_ is the learning rate. This ensures that the network learns to correctly categorize the stimuli (i.e., the pattern of Sensory unit activity, **x**(*t*)) using the output, Category units **y**(*t*). As with Amygdala learning, the categorization learning proceeded in tandem with the relevance learning.

### Learning to ignore and blocking

The first set of simulations tested the network’s ability to learn to ignore irrelevant stimuli and engage in blocking. These were both run using *dt* = 20 ms, which was selected to be just long enough prevent Poisson noise from affecting learning. At the beginning of the simulations, a 60 s adaptation period without stimulus allowed weights to adjust to the randomly-selected baseline input activity levels. All pharmacological simulations were implemented after adaptation.

#### Learning to ignore

The US times were generated with an inter-trial-interval uniformly sampled between 20 and 30 seconds. A relevant stimulus (CS+) was always presented at a fixed interval before the US, while the irrelevant stimulus (CS0) was presented at random times, and thus uncorrelated to the US. The interval between CS+ and US was set to 100 ms for most simulations, but this was immaterial to the learning algorithm (see below). CS+ and CS0 were simulated as 200 ms periods during which the firing rate of a pre-determined set of Sensory units (10%) was raised to *ψ*^*on*^.

The network was capable of learning the relevance of a temporally offset CS+ because *β*(*t*) integrated signals across time-steps with a discounting factor. However, we should note that the temporal difference learning algorithm we used here ultimately corresponds to a TD-λ(0) algorithm [39], which was why it was important to have some overlap between the CS+ and US. With eligibility traces (i.e. λ > 0) it is possible to learn with longer delays between CS+ and the US, including delays that lead to no overlap (see S1 Fig).

#### Blocking

The blocking protocol used the same parameters as that of the previous paradigm, with the exception that there were four phases of stimulus exposures: 1) a pre-exposure phase, in which both CSs (named CS-A and CS-B) were presented 50 times *without* the US (inter-trial-intervals were decreased to 10-15 s to reduce runtime), 2) a conditioning phase, in which CS-A was presented 50 times paired with a US, 3) a blocking phase, in which the CS-B was presented 50 times simultaneously with the CS-A, paired with an US, 4) a test phase, in which the CS-A and CS-B were presented independently 10 times in the absence of an US. To reduce run-times, the inter-stimulus intervals were also reduced to between 10 and 15 seconds. The use of pre-exposures was guided by protocols used in relatively recent work examining functions of frontal cortex regions [113, 114].

#### Tests of inhibitory disruptions in different model versions

The effects of inhibitory connection strength changes on learning to ignore and blocking were assessed using different model versions. The model versions differed with respect to which synapses were plastic: **W**^*x*→*I*^, **W**^*x*→*E*^, or **W**^*I*→*E*^, see Eqs (3), (4), and (21) above. The purpose of the test was to evaluate whether disruptions to inhibitory systems correspondingly disrupt learning to ignore and blocking, as has been hypothesized to take place in schizophrenia. Inhibitory disruptions were made by reducing the degree to which excitatory units could respond to the inhibitory units by 10%:

$$\phi^{E}\left(t\right)=0.9\frac{W^{x\to E}x\left(t\right)+b^{E}}{W^{I\to E}I\left(t\right)}+0.1\left(W^{x\to E}x\left(t\right)+b^{E}\right)$$ (25)

$$E(t)∼Poisson(dt\phi^{E}\left(t\right))$$ (26)

### Simulation of experimental data: Pharmacological effects on latent inhibition

Piantadosi and Floresco (2014) [43] demonstrate the effect of GABA-A antagonists on latent inhibition. Latent inhibition refers to the classic behavioral phenomenon whereby it is harder to associate a familiar stimulus (one that a subject has been pre-exposed to) with a reinforcer [45]. Latent inhibition is also known to be disrupted in schizophrenia [46, 47, 48, 49]. As shown in Fig 5A–5D, a protocol was created that matched the one used in rats (see also [115, 116]). For processing time purposes, the stimulus and inter-stimulus times used in the original were reduced by a factor of 5. The protocol began with a 60 s adaptation period, followed by three phases: 1) a pre-exposure phase, in which the network was presented with the conditioning stimulus (CS) 30 times (10% of input units, 6 s long, inter-stimulus interval of 6 s), 2) a conditioning phase, in which the CS was presented simultaneously with foot shock (*u*(*t*) = 1), and 3) a test phase, in which the CS was presented by itself 4 times. The protocol was performed on three pairs of network models, with each pair including one network given pre-exposures and one that was not given pre-exposures. The three pairs simulated the treatment groups used in the original study: animals treated with saline were simulated without any modification to the network, treatment with GABA-A antagonist during conditioning were simulated using a 20% reduction in inhibition, according to Eq 25 during the conditioning phase, and treatment with antagonist during testing were simulated with the same disruption during the testing phase. Conditioned fear responses were measured as the maximal response of amygdala units, averaged across all timesteps during CS presentation.

### Simulation of experimental data: Optogenetic effects on fear expression

Recent work by Courtin et al. [44] found that inhibition of PV+, fast-spiking neurons in the mouse mPFC can evoke fear responses, while excitation of the same neurons can decrease fear responses. The protocol used in that study was presently simulated as precisely as possible (Fig 6A and 6B), using all of the same parameters as used in the latent inhibition design. To simulate optical inhibition of PV+ cells, a 20% reduction in inhibition were implemented, similar to Eq 25. During the pre-conditioning phase, this reduction in the inputs was applied for 250 ms intervals separated by 860 ms (equivalent to 0.9 Hz stimulations, as in the original study). This was followed by a conditioning phase, in which a 6 s CS+ was paired with footshock (i.e., the firing rate of input units coding for the CS was set to *ϕ*^*on*^ and *u*(*t*) = 1). As in the previous protocol, all stimulus and inter-stimulus times were decreased from the original study by a factor of 5. One change from the original protocol is that the 1 s US presentation used in the original study was lengthened to the entire CS period. We justify this change based on an assumed difference between real brains and the model: whereas in the brain, activity and plasticity are likely regulated by change, such as the onset or offset of a stimulus, the model treats each time point equivalently. Thus, the period during which the CS is on but US is off will extinguish the associations learned during their concurrence. The CS–US pairings were presented 12 times with an inter-trial interval of 4-30 s. The conditioning phase was followed by an extinction phase, in which the CS was again presented 12 times with the same inter-trial interval, followed in turn by a series of CS presentations accompanying the 40% reduction in **W**^*x*→*I*^ values. To test the effect of inhibitory activation during a conditioned CS, the same conditioning protocol was used, but was followed by presentations of the CS accompanying increases to inhibitory unit activity. We found that only a 10% increase in **W**^*I*→*E*^ was necessary to elicit changes approximating those observed in the original study.

### Pairing of relevance-learning with classification learning

To examine the ability of the network to carry both the salience signal and the other information simultaneously (i.e. to multiplex the salience signal with other signals) simulations were run wherein the feedforward excitatory weights (**W**^*x*→*E*^ and **W**^*E*→*y*^) were trained to perform categorization of the inputs, **x**(*t*), while the excitatory weights onto the inhibitory unit, **W**^*x*→*I*^, were trained according to the relevance algorithm described in Eqs (3) and (4) (Fig 7 & S4 Fig). To do this, each of the ten stimuli (described in Stimuli) was presented in a fixed order for 200 ms, and this 2 s sequence (considered 1 epoch) was repeated 50 times, leading to a total simulation time of 100 s. (Note that not all epochs are presented in Fig 7 & S4 Fig, as the learning converged quickly).

### Model code

All code was written in Matlab (Mathworks Inc.), using the Statistics Toolbox. The code can be downloaded for free from https://github.com/jordan-g/Irrelevance-by-Inhibition and used to generate all of the data presented in the paper.

## Supporting information

S1 Fig Learning with delays between CS+ offset and US onset. (**A**) Average Cortex excitatory unit activity (lower plots) and inhibitory unit activity (upper plots) when the offset of CS+ precedes the onset of the US by 100 ms (note the gap between the green and gray blocks at the bottom). This effect was achieved by using *γ* = 0.98 and an eligibility trace for each synapse with a decay factor of λ = 0.8. This corresponds to a TD-λ(0.8) algorithm [39]. See the code online for the specific implementation of the eligibility trace during learning (https://github.com/jordan-g/Irrelevance-by-Inhibition). (**B**) Averaged excitatory unit (left) and inhibitory unit (right) responses to the CS+ (green) and CS0 (orange) across presentations, as compared with non-stimulus periods (black line).
(TIFF)

S2 Fig Firing-rate and weight distributions following learning to ignore training. (**A**) Firing-rate distributions across the **E**(**t**) population during the simulation. Time-bins were 200 ms long. (**B**) Synaptic weight distributions for the **W**^*x*→*I*^ weights following learning to ignore training.
(TIFF)

S3 Fig Demonstration of learning to ignore with multiple inhibitory units (500 inhibitory units). (**A**) Average Cortex excitatory unit activity (lower plots) and average cortex inhibitory unit activity (upper plots) at simulated, 20 ms time steps in response to unlearned stimuli (left side) compared with the end of a series of repeated presentations (right side). As with the simulations where only a single inhibitory unit was used, excitatory responses were initially high to both stimuli, but after learning they increased only in response to the CS+, demonstrating the network to treat the CS0 as less relevant. (**B**) Averaged excitatory unit (left) and averaged inhibitory unit (right) responses to the CS+ (green) and CS0 (orange) across presentations, as compared with non-stimulus periods (grey line). Learning took place over the first 20 trials, after which excitatory responses to the CS0 plateaued to the same level as excitatory responses to untrained inputs. This was due to increased inhibitory responses to the CS0 across the inhibitory population. (**C**) Salience responses (*S*(*t*)) to the CS+ relative to the CS0 during final presentations are plotted for both control conditions and in simulations of inhibitory dysfunction (means ± STD across 30 model runs). Learning was impaired with inhibitory neuron disruption only in the inhibitory neuron plasticity model (**W**^*x*→*I*^).
(TIFF)

S4 Fig Multiplexed stimulus category and relevance codes with multiple rewarded stimuli. (**A**) Diagram illustrating modified model that included both the mechanisms described above for relevance learning (on ***W***^**x→I**^ synapses) in addition to mechanisms learning an output vector that matches categories presented as input (backpropagation algorithm applied to the ***W***^**E→y**^ and ***W***^**x→E**^ synapses). As illustrated by bottom boxes, three of ten stimuli presented to the network were rewarded. (**B**) Average excitatory unit responses to the three rewarded (green) and seven unrewarded stimuli (orange) over time. The network quickly learns to respond more strongly to the rewarded stimuli. (**C**) Performance of the model on input classification. Over the same time that the network learns to discriminate rewarded and unrewarded stimuli, it also becomes capable of matching the output vector to the input. The gray trace shows the percent of presentations that stimuli are correctly classified, which increases quickly before reaching a plateau. The blue trace shows the cross-entropy, an information measure (in natural units of information) based on the output activity distribution that is inversely related to the success of input classification.
(TIFF)

S5 Fig Learned irrelevance—Slowed relevance learning following uncorrelated CS-US presentations. (**A**) Average excitatory unit responses to each presentation of a CS in a learned irrelevance paradigm. One network (red) was exposed to 100 presentations of a CS and an US, where CS and US presentation times were chosen from independent uniform distributions, for a total time of 800 s. The CS and US presentations lasted 200 ms. Afterwards, the CS and US were shown together 100 times at regular intervals of 5 s, with the CS preceding the US by 100 ms. A second network (blue) was only shown the 100 correlated CS-US presentations. Both networks underwent a 60 s adaptation period without stimulus presentations. For these simulations we used the same hyperparameters as in the “learning to ignore” simulations, with the addition of 10 Amygdala units whose hyperparameters were identical to those used in the latent inhibition simulations. The ***W***^**x→I**^ and ***W***^**E→y**^ synapses were updated at every time step. Data shown is mean ± STD from 20 simulations. (**B**) Average Amygdala layer activity during each CS presentation, for the network that was shown uncorrelated CS-US presentations followed by correlated CS-US presentations (red), and for the network shown only correlated CS-US presentations (blue). Note that the blue trace shows a rapid response from the Amygdala, while the red trace takes a few trials to show consistently higher Amygdala responses.
(TIFF)

S1 Data Code to run simulations. Matlab code for running the simulations that generated the data presented in the paper is provided here. Note that the code utilizes the Statistics Toolbox. To run a custom simulation, refer to main_script.m. In order to reproduce any of the figures in the paper, simply run one of the following files instead: learning_to_ignore.m, blocking.m, latent_inhibition.m, fear_expression.m, categorization.m, learned_irrelevance.m.
(ZIP)
