# Supplementary figures and images for: Irrelevance by inhibition: Learning, computation, and implications for schizophrenia
Source: PLoS Comput Biol. 2018 Aug 1;14(8):e1006315. doi: 10.1371/journal.pcbi.1006315 (PMC6089457; doi:10.1371/journal.pcbi.1006315)

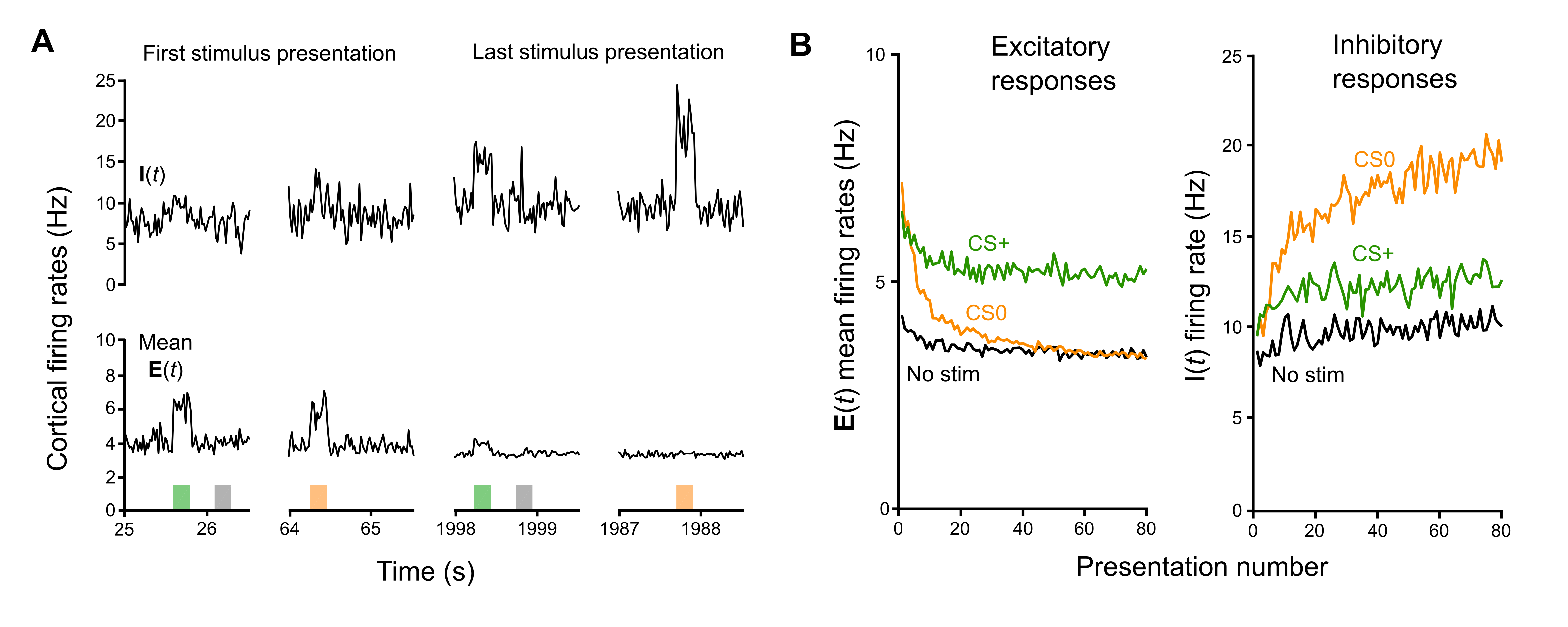

Supplement: S1 Fig — (A) Average Cortex excitatory unit activity (lower plots) and inhibitory unit activity (upper plots) when the offset of CS+ precedes the onset of the US by 100 ms (note the gap between the green and gray blocks at the bottom). This effect was achieved by using γ = 0.98 and an eligibility trace for each synapse with a decay factor of λ = 0.8. This corresponds to a TD-λ(0.8) algorithm [39]. See the code online for the specific implementation of the eligibility trace during learning (https://github.com/jordan-g/Irrelevance-by-Inhibition). (B) Averaged excitatory unit (left) and inhibitory unit (right) responses to the CS+ (green) and CS0 (orange) across presentations, as compared with non-stimulus periods (black line). (TIFF) [file pcbi.1006315.s001.tiff]

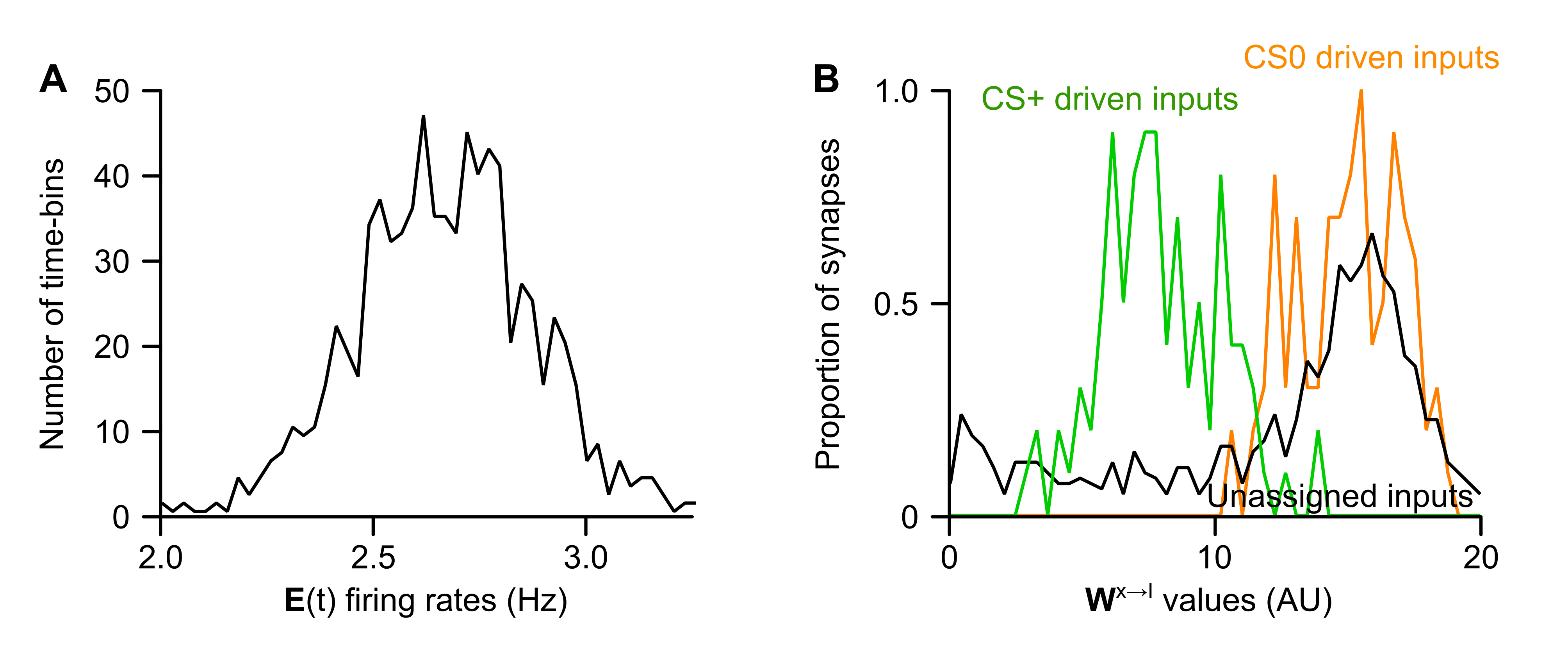

Supplement: S2 Fig — (A) Firing-rate distributions across the E(t) population during the simulation. Time-bins were 200 ms long. (B) Synaptic weight distributions for the Wx→I weights following learning to ignore training. (TIFF) [file pcbi.1006315.s002.tiff]

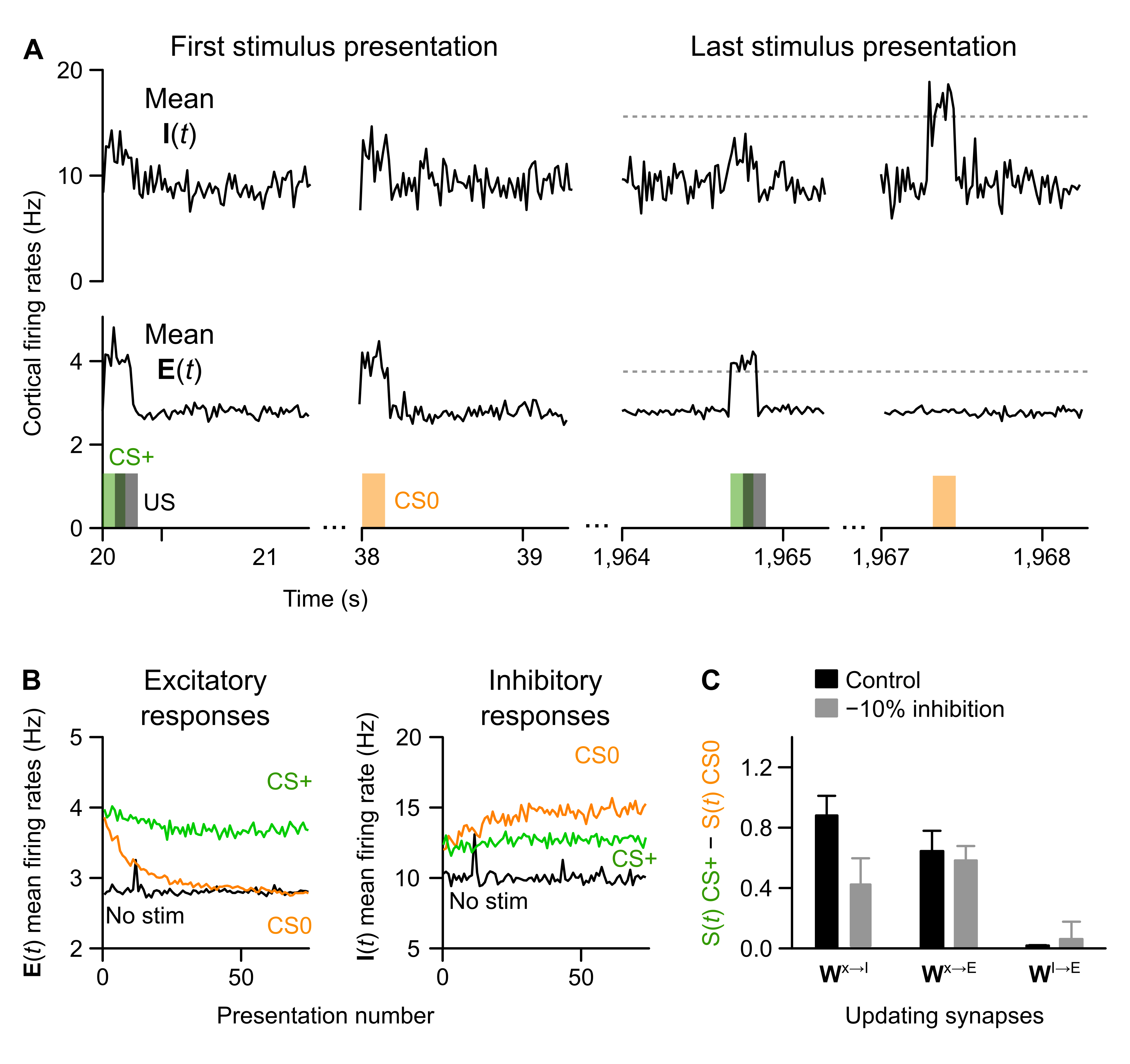

Supplement: S3 Fig — (A) Average Cortex excitatory unit activity (lower plots) and average cortex inhibitory unit activity (upper plots) at simulated, 20 ms time steps in response to unlearned stimuli (left side) compared with the end of a series of repeated presentations (right side). As with the simulations where only a single inhibitory unit was used, excitatory responses were initially high to both stimuli, but after learning they increased only in response to the CS+, demonstrating the network to treat the CS0 as less relevant. (B) Averaged excitatory unit (left) and averaged inhibitory unit (right) responses to the CS+ (green) and CS0 (orange) across presentations, as compared with non-stimulus periods (grey line). Learning took place over the first 20 trials, after which excitatory responses to the CS0 plateaued to the same level as excitatory responses to untrained inputs. This was due to increased inhibitory responses to the CS0 across the inhibitory population. (C) Salience responses (S(t)) to the CS+ relative to the CS0 during final presentations are plotted for both control conditions and in simulations of inhibitory dysfunction (means ± STD across 30 model runs). Learning was impaired with inhibitory neuron disruption only in the inhibitory neuron plasticity model (Wx→I). (TIFF) [file pcbi.1006315.s003.tiff]

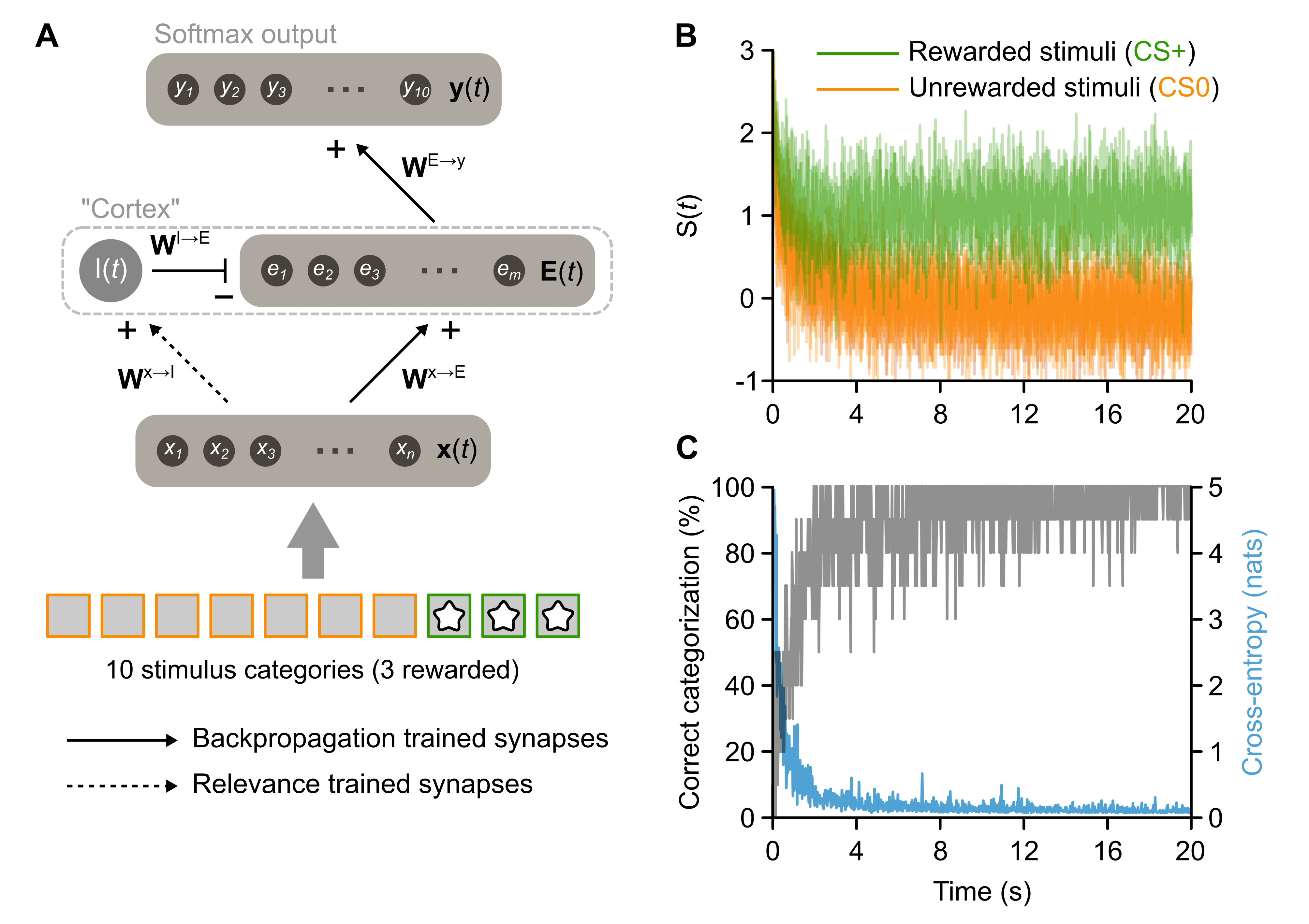

Supplement: S4 Fig — (A) Diagram illustrating modified model that included both the mechanisms described above for relevance learning (on Wx→I synapses) in addition to mechanisms learning an output vector that matches categories presented as input (backpropagation algorithm applied to the WE→y and Wx→E synapses). As illustrated by bottom boxes, three of ten stimuli presented to the network were rewarded. (B) Average excitatory unit responses to the three rewarded (green) and seven unrewarded stimuli (orange) over time. The network quickly learns to respond more strongly to the rewarded stimuli. (C) Performance of the model on input classification. Over the same time that the network learns to discriminate rewarded and unrewarded stimuli, it also becomes capable of matching the output vector to the input. The gray trace shows the percent of presentations that stimuli are correctly classified, which increases quickly before reaching a plateau. The blue trace shows the cross-entropy, an information measure (in natural units of information) based on the output activity distribution that is inversely related to the success of input classification. (TIFF) [file pcbi.1006315.s004.tiff]

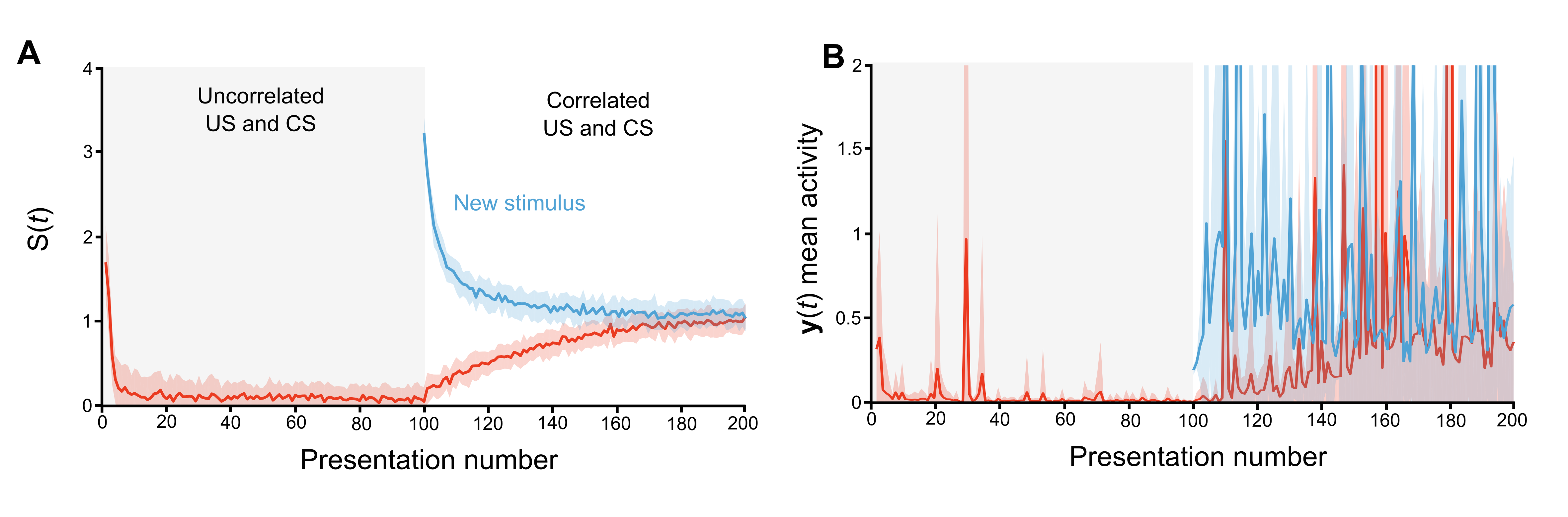

Supplement: S5 Fig — (A) Average excitatory unit responses to each presentation of a CS in a learned irrelevance paradigm. One network (red) was exposed to 100 presentations of a CS and an US, where CS and US presentation times were chosen from independent uniform distributions, for a total time of 800 s. The CS and US presentations lasted 200 ms. Afterwards, the CS and US were shown together 100 times at regular intervals of 5 s, with the CS preceding the US by 100 ms. A second network (blue) was only shown the 100 correlated CS-US presentations. Both networks underwent a 60 s adaptation period without stimulus presentations. For these simulations we used the same hyperparameters as in the “learning to ignore” simulations, with the addition of 10 Amygdala units whose hyperparameters were identical to those used in the latent inhibition simulations. The Wx→I and WE→y synapses were updated at every time step. Data shown is mean ± STD from 20 simulations. (B) Average Amygdala layer activity during each CS presentation, for the network that was shown uncorrelated CS-US presentations followed by correlated CS-US presentations (red), and for the network shown only correlated CS-US presentations (blue). Note that the blue trace shows a rapid response from the Amygdala, while the red trace takes a few trials to show consistently higher Amygdala responses. (TIFF) [file pcbi.1006315.s005.tiff]
